# Transcriptomics and metabolomics reveal the adaption of *Akkermansia muciniphila* to high mucin by regulating energy homeostasis

**DOI:** 10.1038/s41598-021-88397-z

**Published:** 2021-04-27

**Authors:** Xinyue Liu, Fan Zhao, Hui Liu, Yunting Xie, Di Zhao, Chunbao Li

**Affiliations:** 1 Key Laboratory of Meat Processing and Quality Control, Ministry of Education, Key Laboratory of Meat Processing, Ministry of Agriculture and Rural Affairs, Jiangsu Collaborative Innovation Centre of Meat Production and Processing, Quality and Safety Control, Meat Production, College of Food Science and Technology, Nanjing Agricultural University, Weigang 1#, Nanjing, 210095 People’s Republic of China; 2 National Center for International Research on Animal Gut Nutrition, Nanjing Agricultural University, Nanjing, 210095 People’s Republic of China

**Keywords:** Biochemistry, Microbiology

## Abstract

In gut, *Akkermansia muciniphila* (*A. muciniphila*) probably exerts its probiotic activities by the positive modulation of mucus thickness and gut barrier integrity. However, the potential mechanisms between *A. muciniphila* and mucin balance have not been fully elucidated. In this study, we cultured the bacterium in a BHI medium containing 0% to 0.5% mucin, and transcriptome and gas chromatography mass spectrometry (GC–MS) analyses were performed. We found that 0.5% (m/v) mucin in a BHI medium induced 1191 microbial genes to be differentially expressed, and 49 metabolites to be changed. The metabolites of sorbose, mannose, 2,7-anhydro-β-sedoheptulose, fructose, phenylalanine, threonine, lysine, ornithine, asparagine, alanine and glutamic acid were decreased by 0.5% mucin, while the metabolites of leucine, valine and N-acetylneuraminic acid were increased. The association analysis between transcriptome and metabolome revealed that *A. muciniphila* gave strong responses to energy metabolism, amino sugar and nucleotide sugar metabolism, and galactose metabolism pathways to adapt to high mucin in the medium. This finding showed that only when mucin reached a certain concentration in a BHI medium, *A. muciniphila* could respond to the culture environment significantly at the level of genes and metabolites, and changed its metabolic characteristics by altering the effect on carbohydrates and amino acids.

## Introduction

*Akkermansia muciniphila* (*A. muciniphila*) is a Gram-negative bacterium and belongs to the phylum *Verrucomicrobia*^1–4^. Its abundance in the gut is largely related to age, diet and health status of the host. This bacterium begins to colonize human intestinal tract at the early life, and its abundance increases to healthy adult levels (10^8^ cells/g) within a year, but falls in old ages^5^. Higher abundance of *A. muciniphila* is associated with a healthier metabolic status in overweight or obese subjects^6^. Previous studies showed that the abundance of *A. muciniphila* is inversely related to host metabolic diseases^7–10^, indicating protective and anti-inflammatory potentials.

In the gut, mucin-degrading bacteria reside in the loose mucus layer, where *A. muciniphila* utilizes mucin as nutrient for growth^11^. Mucus is an important defense barrier and comprises of complex polysaccharide-proteins (i.e., mucins) with a polymeric structure, which is secreted by goblet cells in epithelial tissue. Mucus comprises of a firm inner layer and a loose outer layer. The thickness of the mucus layer increases from 125 μm at the jejunum to 830 μm at the colon in the gastrointestinal tract^12,13^. The differences in mucus thickness reflect the protective effects of the mucus. Patients with ulcerative colitis have a thinner mucus layer in the tissue, while patients with Crohn’s disease have normal or even thicker mucus layer in the colon tissue^14,15^. Similarly, the thickness of the mucus layer is strongly related to the abundance of *A. muciniphila* in the gastrointestinal tract. Intake of arabinoxylans may increase colonic mucus thickness and the abundance of *A. muciniphila* in humanized rats^16^. High-fat diet would reduce the abundance of *A. muciniphila*^17^, while daily gavage of *A. muciniphila* can restore mucus layer thickness upon high fat diet^18^. In aged mice the thickness of the colonic mucus layer was reduced about sixfolds relative to young mice^19^. Thus, mucus alterations appear to characterize gut diseases in response to intestinal microbes (including *A. muciniphila*) or host-derived inflammatory mediators. However, the exact mechanisms by which *A. muciniphila* exerts the beneficial impact on heath associating with mucus have not been fully elucidated.

Mucins are large extracellular glycoproteins and consist of protein backbones decorated with a variety of carbohydrate chains, which are directly correlated with the protective properties of mucus gel. The mucin protein core contains highly glycosylated regions comprising of 80% carbohydrates primarily of N-acetylgalactosamine (GalNac), N-acetylglucosamine (GlcNac), fucose (Fuc), galactose (Gal) and N-acetylneuraminic acid (Neu5Ac) and traces of sulfate (SO_4_^2−^) and mannose (Man), while the peptide backbone is rich in threonine, serine, cysteine and proline^20,21^. *A. muciniphila* could directly utilize glycans and amino acids from the peptide backbone in mucins as a nutrient. Stable isotope labeling and in situ hybridization imaging revealed that *A. muciniphila* has the ability to use host-protein derived amino acids in vivo^22^. *A. muciniphila* exerted its metabolic properties attributed to the mucin-degrading enzymes. The whole genome of *A. muciniphila* has been sequenced, and it indicates that many genes encode mucin-degrading enzymes, including glycosidases and sulfatases^23^. These enzymes play an important role in mucin degradation, which release glycans for the growth of the mucin-degrading bacteria and affect other residents in the gut. Hexosaminidase hydrolyzes terminal non-reducing N-acetyl-d-hexosamine residues in N-acetyl-beta-d-hexosaminides^24,25^. The β-galactosidase hydrolyzes β-d-galactosyl residues from the non-reducing ends of glycoconjugates^26^. The α-l-fucosidase liberates terminal α-linked l-fucose from the oligosaccharides of various glycoconjugates including mucin glycoprotein^27^. The α-N-acetylglucosaminidase is involved in sulfate degradation^28^. The α-galactosidase hydrolyzes the terminal alpha-galactosyl moieties from glycolipids and glycoproteins^29^. The α-glucosidase hydrolyzes non-reducing terminal α-d-glucose residues with release of α-d-glucose^30^.

The in vitro cultivation method has been applied to study the responses of *A. muciniphila* to different levels of mucin. Ottman et al. applied a genome-scale metabolic model to predict how *A*. *muciniphila* utilized mucin-derived monosaccharides, including fucose, galactose, and N-acetylglucosamine in a minimal growth medium^31^. *A*. *muciniphila* prefer to grow in a special medium (a medium containing GlcNAc or GalNAc) or a BHI medium. In a BHI medium, it is not fully understood how *A. muciniphila* adapts to different physiological conditions varying with mucin contents.

In this study, we integrated transcriptomic and metabolomic analyses to explore the responses of *A. muciniphila* to mucin addition in a BHI medium, and its underlying mechanism was interpreted. To our knowledge, this is the first study that has evaluated metabolites profiles of *A. muciniphila* and mucin using transcriptome and GC–MS in vitro, and it will be an intensive research for the physiology of *A. muciniphila* to exert its probiotic functions in gut.

## Results and discussion

### Mucin addition affected growth rate and morphology of *A. muciniphila*

**Figure 1:**
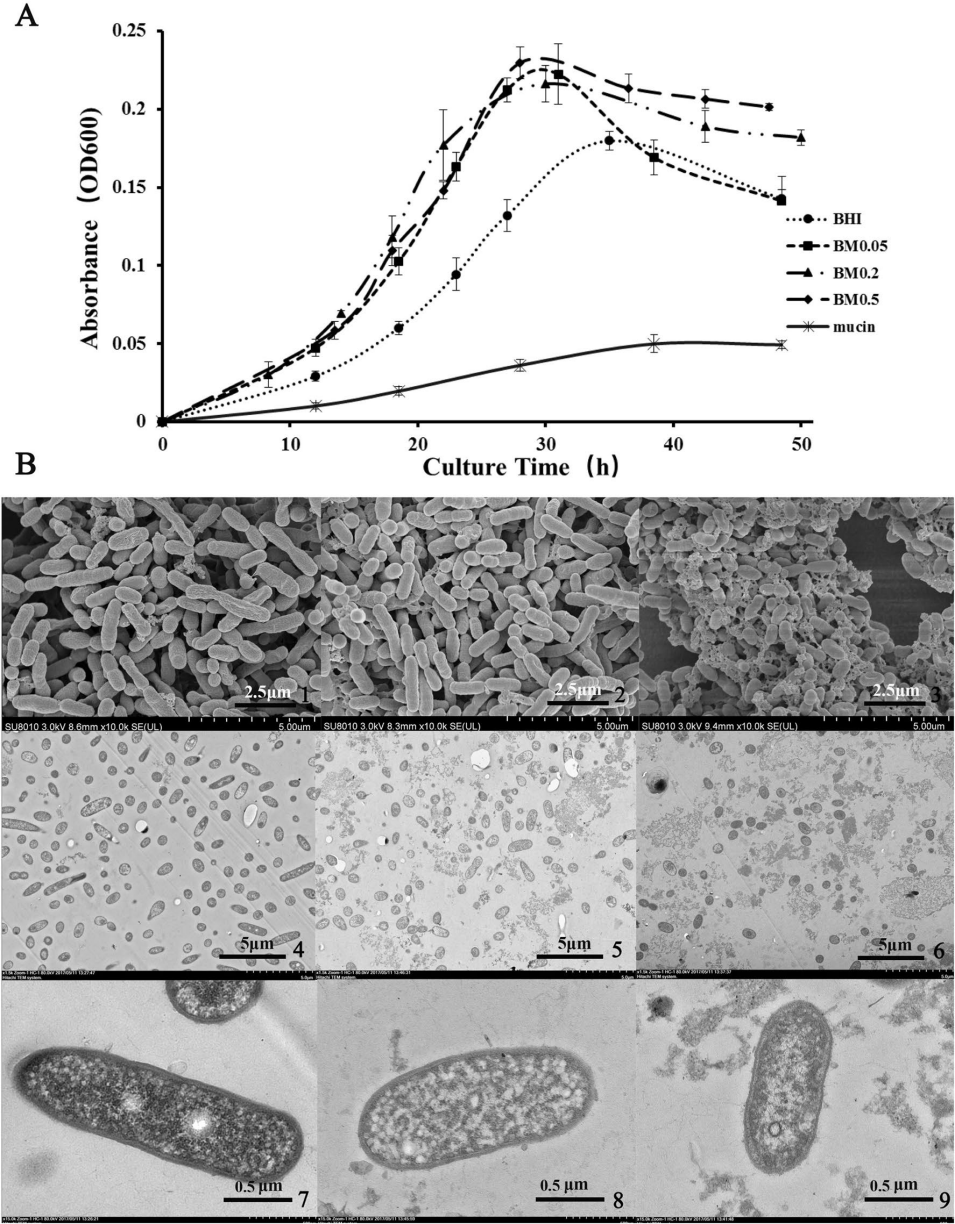
Mucin-dependent changes of *A. muciniphila*. (**A**) Growth curves; (**B**) Scanning and transmission electron microscopy images of *A. muciniphila* growing in BHI, BM0.05 and BM0.5, respectively. Among them, numbers 1, 4 and 7 represent groups without mucin; numbers 2, 5 and 8 represent groups with 0.05% mucin; numbers 3, 6 and 9 represent groups with 0.5% mucin.

Bacterial growth curves indicated a mucin-dependent pattern for *A. muciniphila*. The absorbance changed slightly when *A. muciniphila* was grown in a mucin medium (Fig. 1A). This might be associated with the lack of rumen fluid in our mucin medium. Instead, this bacterium could grow better in a BHI medium alone or containing mucin. Compared with control (BHI-only), it took a shorter time for the bacterium in the BM0.5 group to reach the maximum abundance (35 h for the control group vs. 28 h for the BM0.5 group, Fig. 1A). The times for the BM0.05 and BM0.2 groups were 31 h and 30 h, respectively. In our previous in vitro cultivation studies, two different proteins (soybean and chicken proteins) were added as additional nitrogen source for *A. muciniphila* growth in a BHI medium, and we found that the OD_600_ values of the chicken-protein-supplied group were higher than those of the BHI-only group^32^. *A. muciniphila* in mucin-added BHI medium had shorter time to reach the maximum abundance, confirming the findings of Ottman et al.^31^ that the growth rate of *A. muciniphila* was increased greatly when a minimal medium was mixed with 0.25% mucin and glucose. It was notable that 0.5% mucin increased microbial absorbance in the present study and Ottman et al.^31^. Previous studies indicated that *B. thetaiotaomicron* showed a diauxic growth curve in a porcine mucin O-glycans medium^33^. However, mucin addition to the BHI medium induced *A. muciniphila* to grow in a unimodal growth curve in the present study. Scanning electron microscopy showed that the size of *A. muciniphila* was smaller when it was grown in a BHI medium containing 0.5% mucin compared with control or lower levels of mucin (Fig. 1B). van der Ark also observed that *A. muciniphila* had a smaller size when growing in a basic mucin medium compared with those being cultivated in a soy protein medium^34^. *A. muciniphila* growing in the BM0.5 medium had smaller diameter than growing in the BHI-only medium (Fig. 1B). Most inclusions were considered the reserves for nutrients or energy in response to nutrient imbalance^35^. According to these observations, we speculate that mucin addition in BHI medium promotes the growth and division of *A. muciniphila*, as the faster the cell division rate, the smaller the cell morphology.

### Transcriptomic analysis of *A. muciniphila* in response to mucin addition in BHI medium

Morphological and molecular changes may occur to *A. muciniphila* when the medium is changed. And thus whole-genome sequencing was performed to establish a set of reference sequences for transcriptome analysis (Supplementary Fig. S1, Fig. S2, Table S1 and Table S2). Transcriptome sequencing generated 15 to 34 million raw reads per sample, and there were 18,383,453, 18,396,179, 23,347,600 and 19,791,598 clean reads in the BHI-only, BM0.05, BM0.2 and BM0.5 groups, respectively. The Q20 values (sequencing error rate < 1%) were greater than 95.66%. The average GC content of four groups were 57.94%, 57.43%, 56.39% and 51.69%, respectively and the rRNA reads accounted for less than 0.35% of raw reads in the studied samples (Supplementary Table S3). These results demonstrated that the quantity and quality of sequencing data were sufficient to ensure accurate sequence assembly and adequate transcriptome coverage.

**Figure 2:**
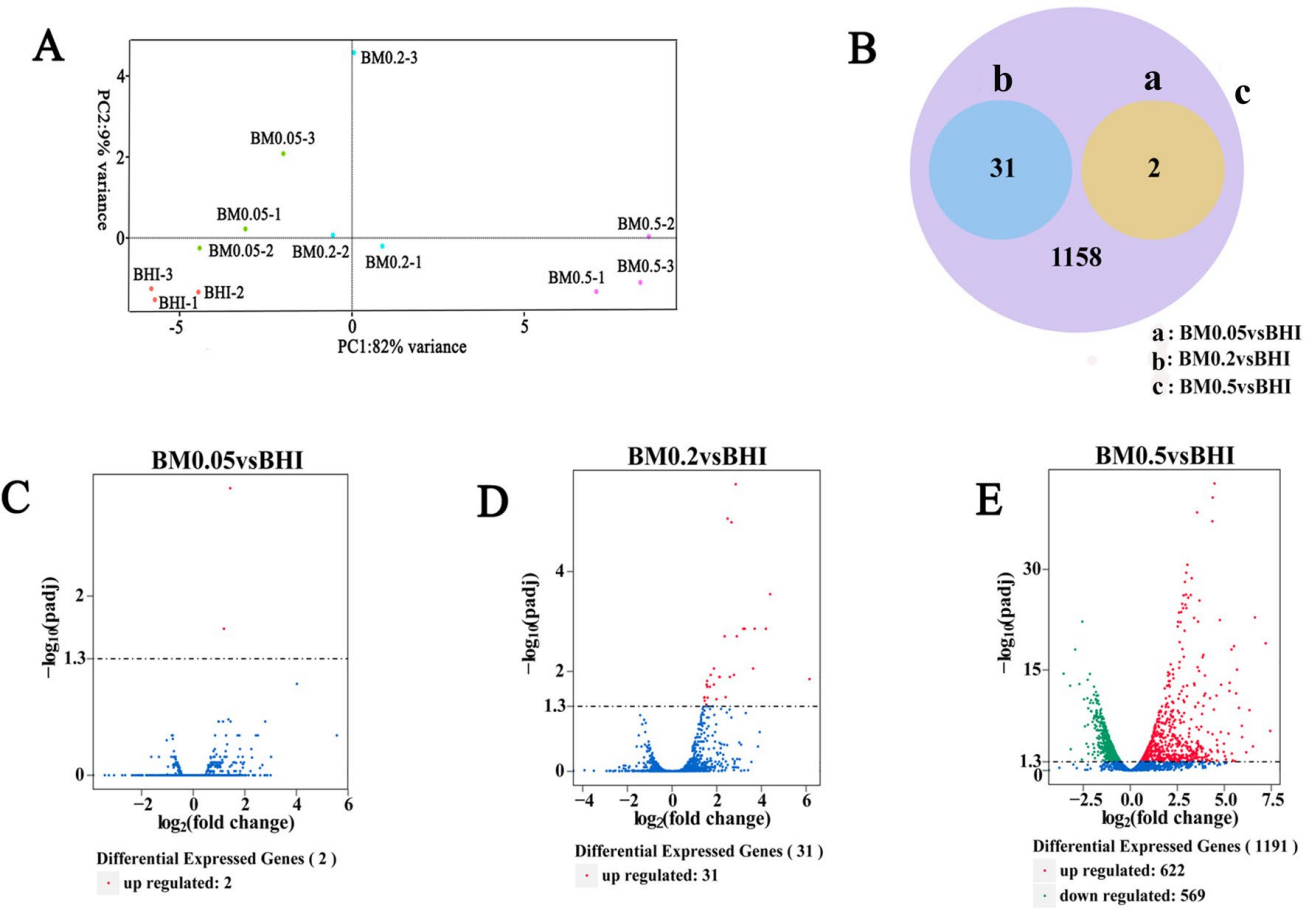
DEGs in *A. muciniphila* in response to mucin. (**A**) scatter plot of principal component analysis; (**B**) Venn diagram of DEGs; (**C**) Volcano plot of DEGs in BM0.05; (**D**) Volcano plot of DEGs in BM0.2; (**E**) Volcano plot of DEGs in BM0.5. Red spots represent upregulated genes, and green spots represent downregulated genes.

Principle component analysis (PCA) of transcriptomic data revealed that the first two principal components (PCs) accounted for 91% of the total variance. The PC1 mainly reflects the differences between the control and 0.5% mucin groups, indicating a significant impact of mucin addition on the growth of *A. muciniphila* (Fig. 2A). Venn diagram and volcano plots indicated that higher mucin addition resulted in higher number of DEGs (Fig. 2B–E).

### Functional analysis of DEGs

**Figure 3:**
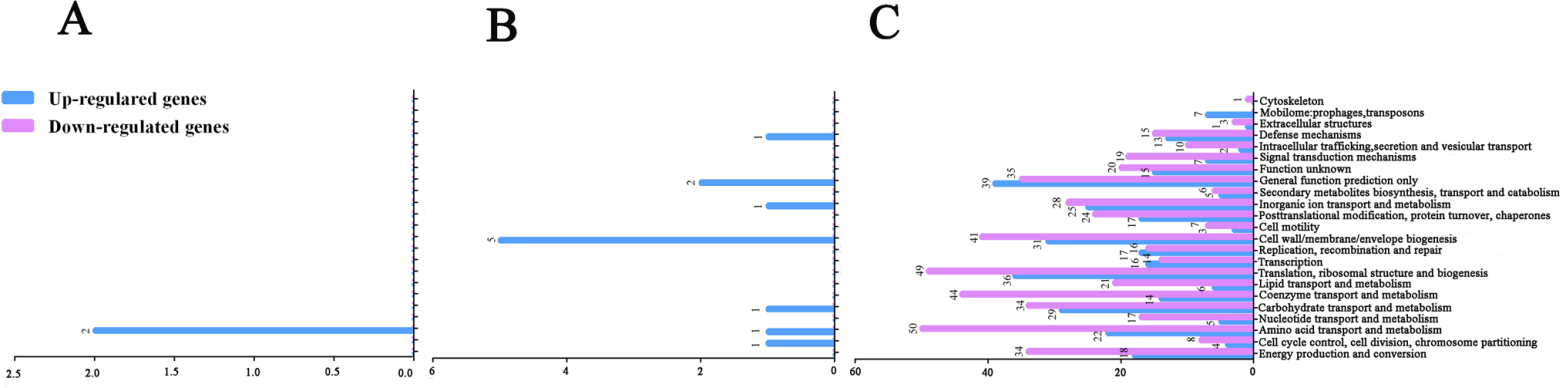
COG annotations of DEGs. (**A**) to (**C**) DEGs in BM0.05, BM0.2 and BM0.5 groups in the COG database, respectively.

The number of DEGs increased as mucin addition to the BHI medium increased. When 0.05% mucin was added, only two genes were upregulated involving amino acid transport and metabolism (Fig. 3A; Supplementary Fig. S3, Fig. S4A, Table S4). In the BM0.2 group, 31 DEGs were annotated to the categories of “cell wall, membrane and envelope biogenesis”, “ATPase activity”, and “nucleoside-triphosphatase activity” (Fig. 3B; Supplementary Fig. S3, Fig. S4B). In the BM0.5 group, 622 DEGs were upregulated and 569 DEGs were downregulated, which were primarily related to cell growth and energy metabolism. Many of the upregulated genes were indicators of increased growth (“translation, ribosomal structure and biogenesis”, “cell wall, membrane and envelope biogenesis”, “carbohydrate transport and metabolism”, and so on) in the COG database (Fig. 3C). The top GO terms were most enriched in biological process and cell homeostasis that related to mucin degradation (Supplementary Fig. S4C). This finding indicates that 0.5% mucin can largely affect the growth and regulation of biological process in *A. muciniphila.* Seventeen and 12 DEGs were enriched in ribosome and glycan degradation, respectively (Supplementary Table S4), of which a gene encoding α-amylase (Amuc_1812) was upregulated. This enzyme catalyzes the hydrolysis of α-1,4-glucosidic bonds in starch and α-glucans^36^. However, Ottman et al. found that this gene was downregulated in 0.5% mucin and glucose in the minimal medium^31^. The difference between our study and Ottman et al. may be due to the higher glucose abundance in BHI medium^31^. The growth rate of bacteria is affected by many factors, e.g., the cellular machinery of ribosome, RNA polymerase activity, ATP concentration, cell wall integrity, and DNA replication^37^. This explains the upregulation of many DEGs in the ribosome pathway in BM0.5 group.

**Figure 4:**
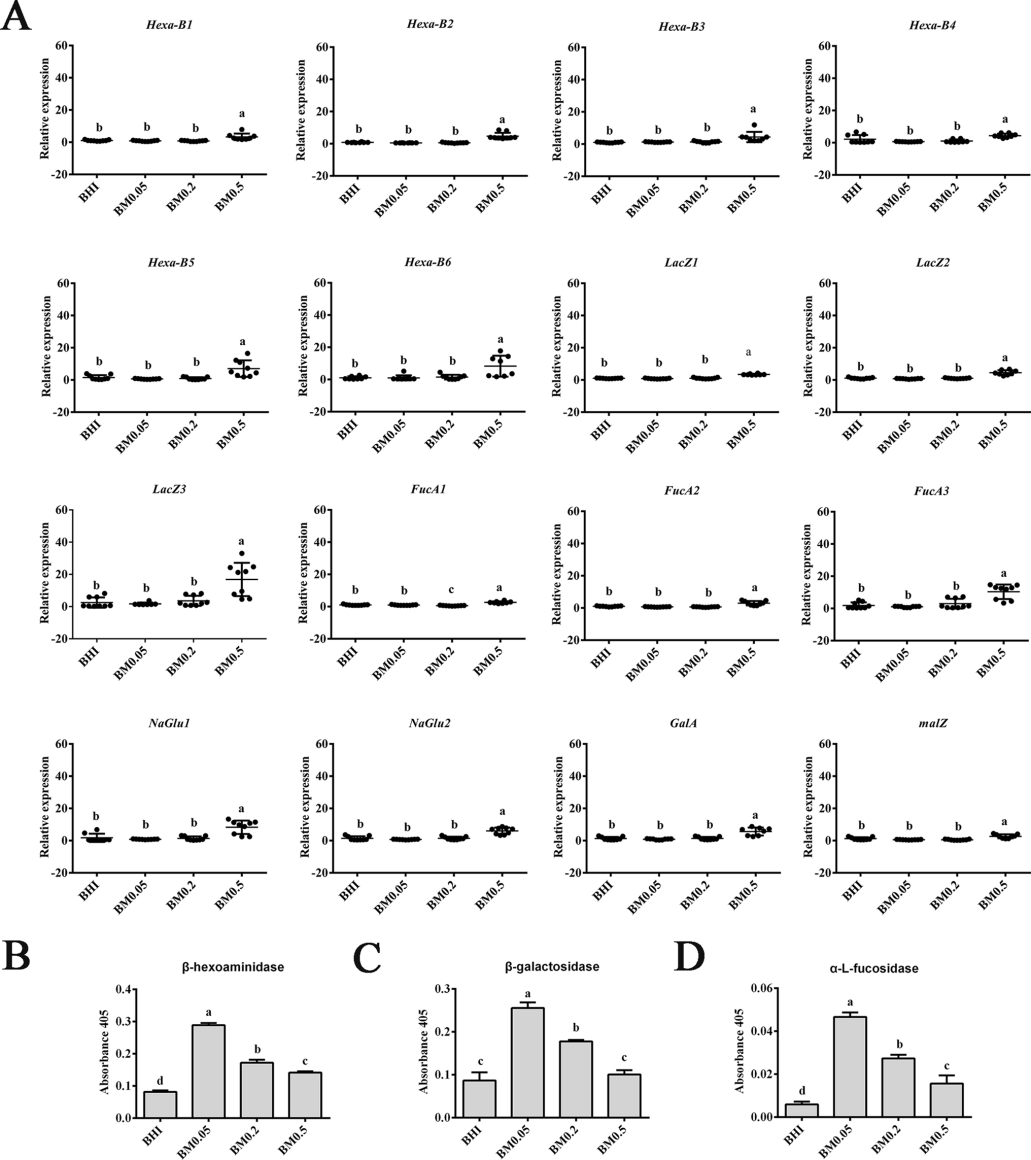
qRT-PCR and activities of mucin degrading enzymes. (**A**) qRT-PCR results; (**B**) and (**C**) Reactions were incubated with 4-nitrophenyl-N-acetyl-β-d-glucosaminide or 4-nitrophenyl-β-d-galactopyranoside in PBS buffer (50 mM, pH 7.0) for 1 h at 37 °C; (**D**) α-l-fucosidase activities were measured by incubation with 2-chloro-4-nitrophenyl-α-l-fucopyranoside in PBS buffer (50 mM, pH 7.0) for 2.5 h at 37 °C. All these enzymatic reactions were stopped by adding 100 μl 1 M Na_2_CO_3_ and the absorbance was read at 405 nm. “a” “b” “c” and “d” indicate significant differences in enzyme activities.

Transcriptomic data indicated that addition of 0.05% mucin may increase ammonia assimilation in *A. muciniphila* by upregulating gene expression of glutamine synthetase and glutamate synthase. Addition of 0.2% mucin may enhance ATPase, cell adhesion, glycan degradation and peptidoglycan biosynthesis. Higher mucin addition (0.5%) upregulated gene expression of hexosaminidases (HexA_B), β-galactosidases (LacZ), α-l-fucosidase (FucA), α-N-acetylglucosaminidase (NaGlu), α-galactosidase (galA) and α-glucosidase (malZ) (Fig. 4A). However, the in vitro activities of β-hexosaminidase, β-galactosidase and α-l-fucosidase increased when the mucin addition increased from 0 to 0.05% but the enzymatic activities decreased greatly when the mucin addition further increased (Fig. 4B–D). We speculate that the inconsistence between in vitro activities and gene expression could be attributed to related glycoside hydrolases in the BM0.5 group that could be attached to mucin and filtered out.

**Figure 5:**
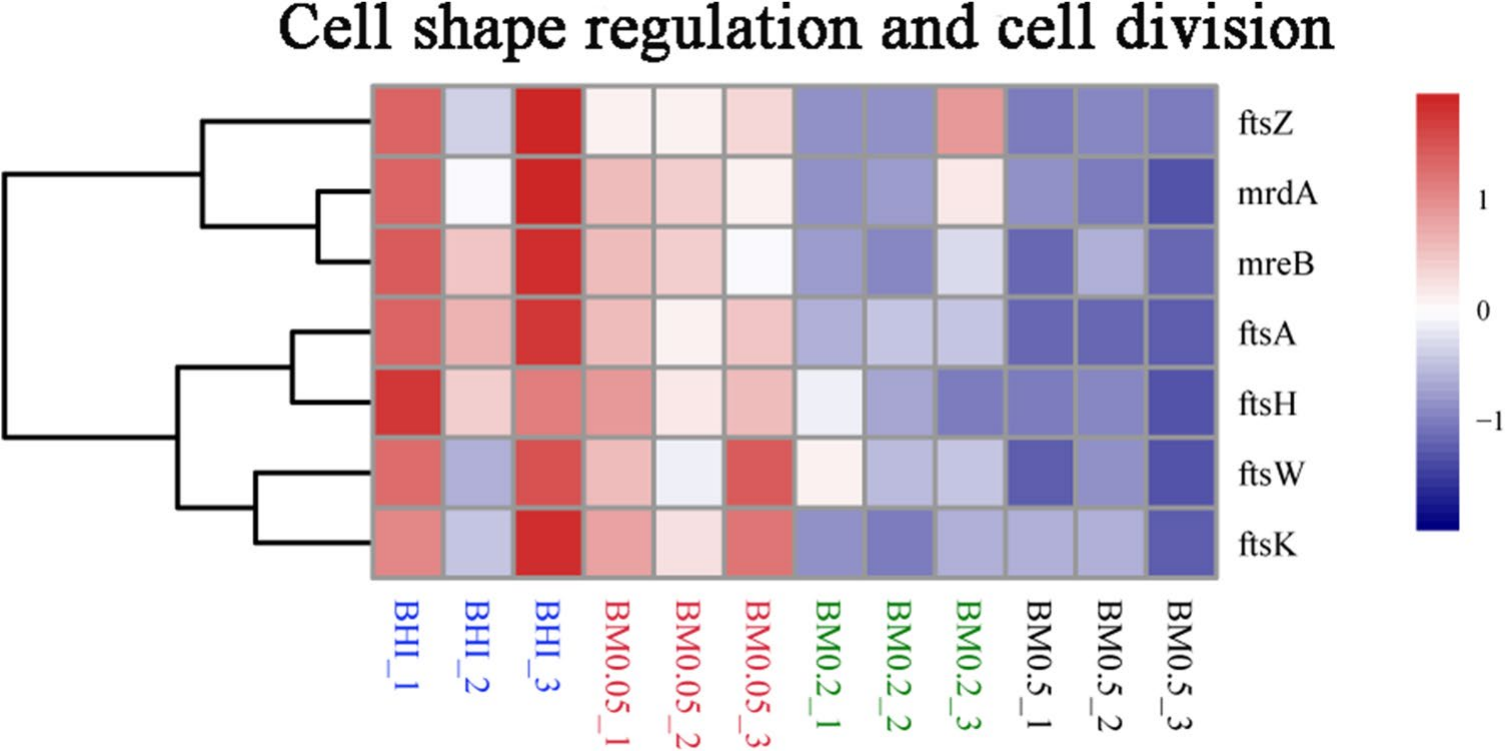
Heatmap analysis of genes related to cell morphology.

DEGs related to DNA replication and cell morphology were listed in heatmap (Fig. 5; Supplementary Table S5). The genes related to the faster growth were up-regulated and the genes related to smaller sizes of *A. muciniphila* were downregulated in the BM0.5 group. In addition, the more abundant carbon source in the BM0.5 group may also contribute to the faster growth of *A. muciniphila*. The cell shape determining protein MreB (Amuc_0540) was downregulated by fourfolds in the BM0.5 group compared with BHI-only group. The mreB is a key gene regulating cell morphology and increasing cell length in *A. muciniphila*^34^.

### Metabolome profiles of *A. muciniphila* in response to mucin addition to BHI medium

To explore the metabolome profiles of *A. muciniphila* when different amounts of mucin were added to the BHI medium, the BHI, BM0.05 and BM0.5 cultures were subject to GC–MS non-target metabolic analysis. An OPLS-DA model showed a good separation of metabolites among the three groups (Supplementary Fig. S5 and Table S6).

**Figure 6:**
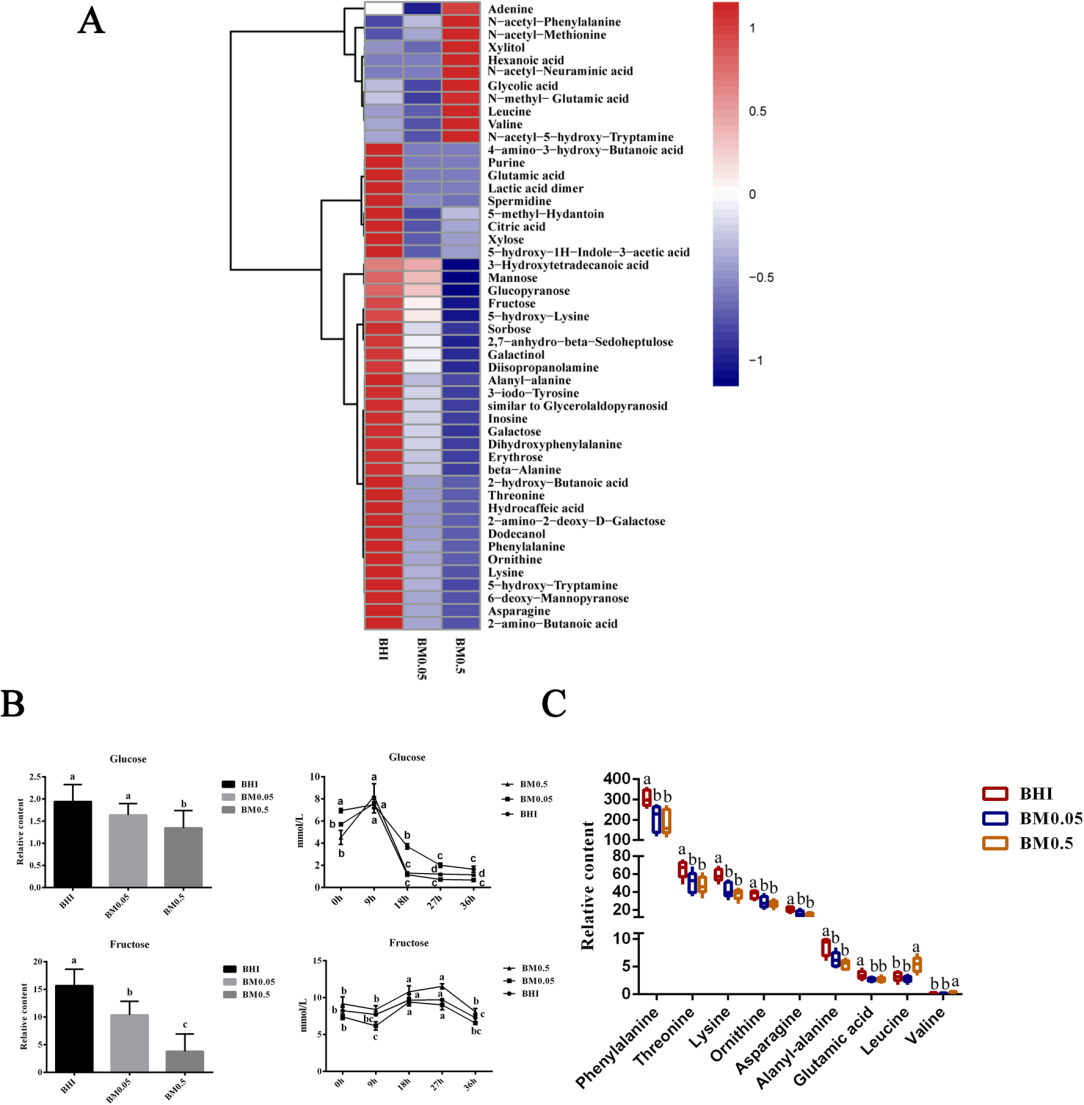
Metabolites in mucin-containing cultures (**A**) Heatmap of metabolites; (**B**) glucose and fructose varying in BHI, BM0.05 and BM0.5 cultures. “a” “b” “c” and “d” indicate significant differences among different time points. (**C**) Relative contents of amino acids (P < 0.05).

**Figure 7:**
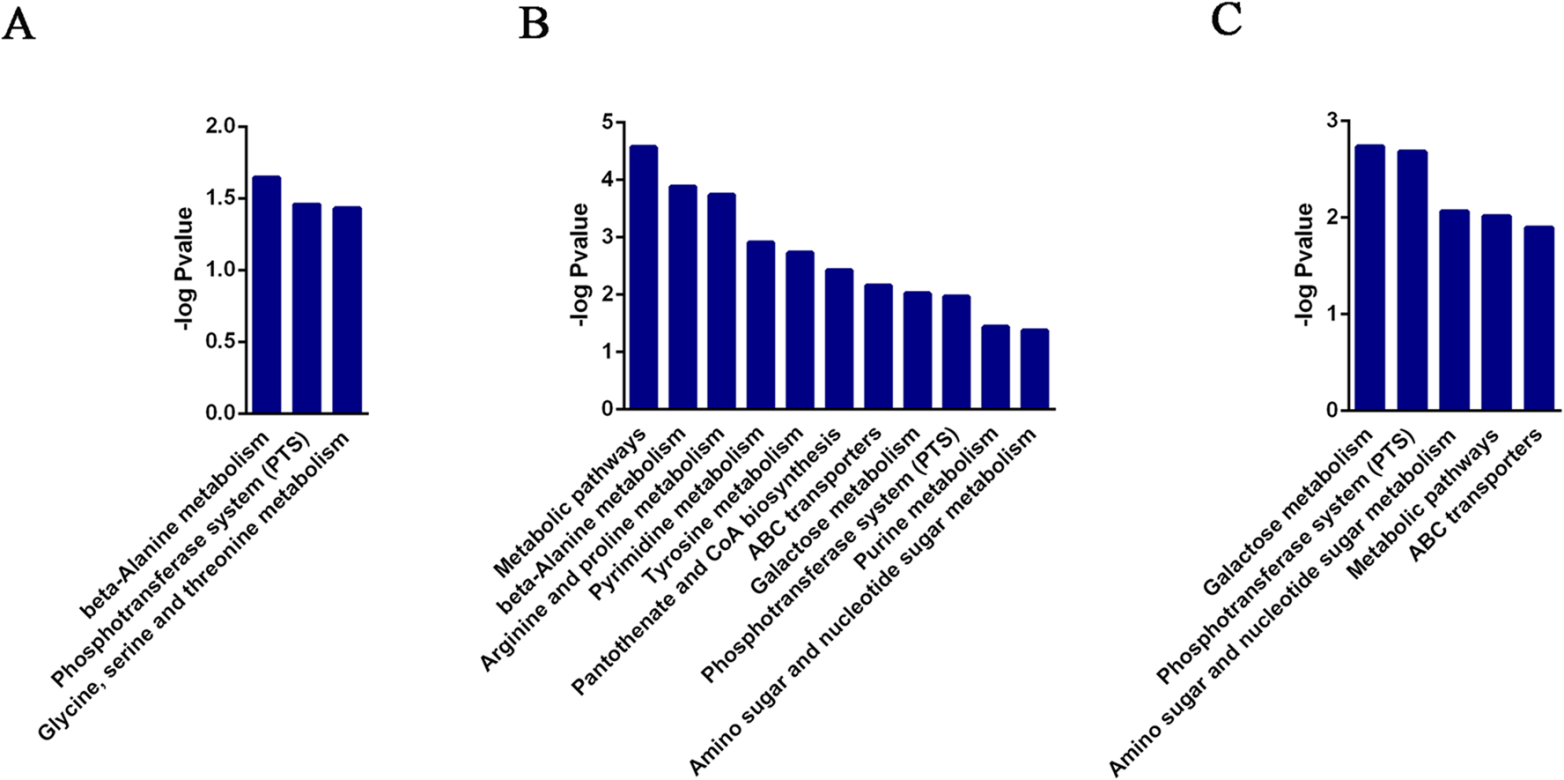
Enrichment of metabolites (P < 0.05, Fold change > 1) in KEGG database. (**A**–**C**) Pathways enriched in the KEGG database between BM0.05 and BHI groups, between BM0.5 and BHI groups, and between BM0.5 and BM0.05 groups, respectively. All the models were analyzed by the OmicsBean.

A total of 161 compounds were identified and analysis of variance showed that 49 compounds of them were significantly different (P < 0.05, Fig. 6A, Supplementary Table S7). The top different compounds were sugars, including sorbose, mannose, 2,7-anhydro-beta-sedoheptulose and fructose. The relative abundances of these compounds decreased when higher mucin was added to the BHI medium (Fig. 6A). This indicates that mucin addition may enhance carbon metabolism of *A. muciniphila*. Mannose is present in mucin and can be used for N-glycan biosynthesis. However, mannosidase was not annotated in the genome of *A. muciniphila*. It is still unknown how mannose is produced and the most important is that bacteria need a balanced carbon–nitrogen ratio in the nutrient-rich conditions^38^. These significantly different metabolites were annotated in KEGG database and related pathways were enriched. Compared with the BHI group, addition of 0.05% mucin upregulated the pathways related to mucin degradation (beta-alanine, glycine, serine and threonine metabolism) and energy metabolism (phosphotransferase system, PTS) (P < 0.05, Fig. 7A). When the mucin addition increased to 0.5%, more pathways were significantly enriched, which are related to mucin degradation (beta-alanine, tyrosine, arginine and proline metabolism, galactose metabolism, and amino sugar and nucleotide sugar metabolism), energy metabolism (PTS), and pantothenate and CoA biosynthesis (Fig. 7B,C). Common to each mucin was a proline-threonine-serine (PTS) domain and the PTS domain was the site of extensive O-glycosylation with carbohydrates accounting for up to 80% of the total mucin mass^39^. It is likely that high concentration of mucin in the BHI medium could stimulate *A. muciniphila* to produce numerous glycoside hydrolases. First, the release of sialic acid from non-reducing ends is an initial step in the sequential degradation of mucins^40^. Second, the mucin pore glycans were exposed to further enzymatic degradation for other glycoside hydrolases (β-hexosaminidase, β-galactosidase and α-l-fucosidase etc.). Finally, the bacteria would enter the PTS pathway for further energy metabolism and promote cell growth^41^.

Metabolome analysis revealed that the relative abundance of glucose and fructose decreased as the mucin addition increased (P < 0.05, Fig. 6B). During the cultivation, the absolute content of glucose showed an increase from 0 to 9 h, but it decreased greatly afterwards (P < 0.05). The absolute content of fructose increased from 0 to 18 h and then declined (P < 0.05). The addition of mucin to BHI medium appeared to accelerate the breakdown of glucose (Fig. 6B). This is because glucose plays an important role in energy metabolism and is the preferred energy source for bacteria. BHI medium contains both glucose and nitrogen source. The three cultures had significantly different relative abundances of phenylalanine, threonine, lysine, ornithine, asparagine, alanyl-alanine, glutamic acid, leucine and valine (P < 0.05, Fig. 6C). In particular, the relative abundance of free threonine decreased as the mucin addition increased in the BHI medium, which probably explains the downregulation of threonine-related DEGs in the BM0.5 group (Supplementary Table S8). Free leucine and valine were highly abundant in the BM0.5 group (P < 0.05), while other free amino acids were more abundant in the BHI-only group (P < 0.05), which could be attributed to the downregulation of DEGs involved in leucine biosynthesis (isopropylmalate dehydrogenase; Amuc_0667, Amuc_0785) and mucin degradation in the BM0.5 group, indicating the existence of a similar mechanism for formation of special amino acids in *A. muciniphila*^42,43^. As shown above, the genes of mucin-degrading enzymes were upregulated by 0.5% mucin addition, but extracellular activities of these enzymes were lower. As an enzymatic product, N-acetylneuraminic acid was highly abundant in the BM0.5 group, which may inhibit the production of other acid substances (Fig. 6A).

### Mechanisms on how *A. muciniphila* responds to high mucin under integrative analysis of transcriptome and metabolome data

To establish a relationship between DEGs and metabolites, several genes and metabolic pathways were listed in Supplementary Table S9. Three pathways were significantly enriched in the BM0.5 group compared with the BHI or BM0.05 groups, that is, amino sugar and nucleotide sugar metabolism, galactose metabolism and ABC transporters. Mucin contains a large amount of amino sugars (GlcNAc and GalNAc), indicating that *A. muciniphila* significantly accelerated its cell growth in the BM0.5 group.

In the amino sugar and nucleotide sugar metabolism pathway, genes encoding amino sugar degrading enzymes, e.g., N-acetylneuraminatelyase, hexosaminidase and glucosamine-6-phosphate deaminase were enriched. Glucosamine-6-phosphate deaminase catalyzes the conversion of glucosamine 6-phosphate to fructose 6-phosphate. The gene encoding UTP-glucose-1-phosphate uridylyltransferase was downregulated, which catalyzes the synthesis of UDP-glucose from UTP and d-glucose 1-phosphate. Mannose-1-phosphateguanylyl transferase catalyzes the conversion of d-mannose 1-phosphate to GDP-mannose. In the galactose metabolism pathway, genes encoding alpha-galactosidase and beta-galactosidase were upregulated, while genes encoding galactokinase were downregulated. This might be related to the decreasing galactinol when the mucin addition increased from 0 to 0.5%, which is a donor of galactose. ABC transporters are widely found in microorganisms and play an important role in nutrient intake^44^.

Except for the above three common metabolic pathways, other four specific metabolic pathways were identified in the BM0.5 group compared with the BHI group (Supplementary Table S10), that is, pyrimidine metabolism pathway, purine metabolism pathway, pantothenate and CoA biosynthesis pathway, and arginine and proline metabolism pathway. The first three pathways are involved in cell growth. In the fourth pathway, the downregulated gene encoding pyrroline-5-carboxylate reductase is related to proline metabolism^45^.

**Figure 8:**
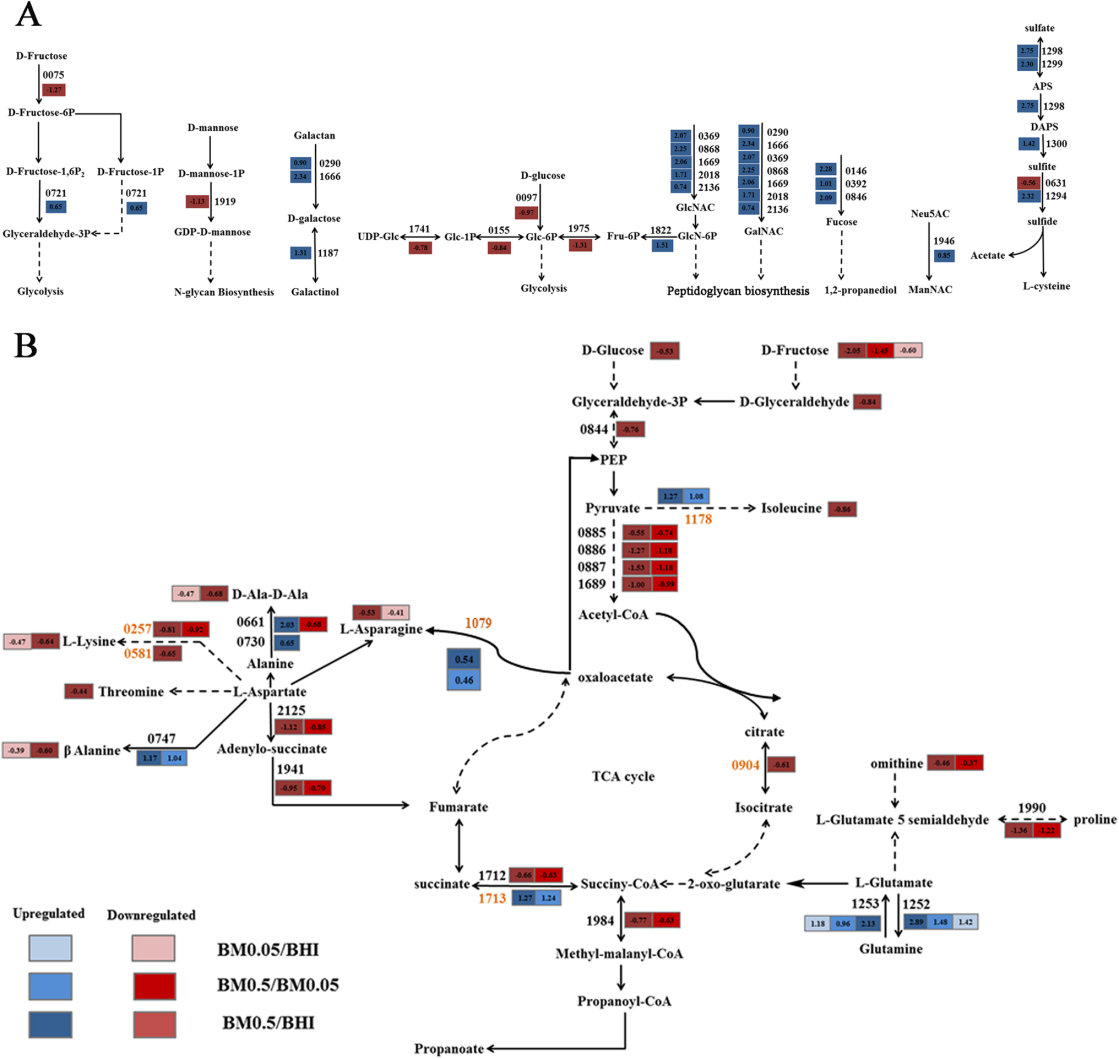
Proposed mechanism of mucin regulating the growth of *A. muciniphila.* (**A**) Pathways regulating the growth of *A. muciniphila* in BM0.5 group. (**B**) Pathway related to mucin-dependent metabolism in *A. muciniphila*. The proposed metabolic pathways were based on Swiss-Prot and KEGG databases. Blue boxes represent upregulated genes or metabolites, and red boxes represent downregulated genes or metabolites. The figures in the boxes represent log_2_fold_changes between BM0.5 and BHI groups. The number on arrows represents the number of DEGs in KEGG database (For example: 0146 represent amu: Amuc_0146). Straight arrows indicate the direct effect between two substances. Dotted arrows indicate multiple steps between two substances. The proposed metabolic pathways are based on KEGG database (http://www.kegg.jp/kegg/kegg1.html).

Based on the above findings*,* we proposed a mechanism on how *A. muciniphila* responded to mucin addition in a BHI medium by combining the existing pathways of *A. muciniphila* in the KEGG database with the achieved omics data in the present study (Fig. 8). High mucin addition induced *A. muciniphila* to overexpress fucosidase, β-galactosidase, hexosaminidase to degrade mucins into fucose, N-acetylgalactosamine and N-acetylglucosamine. The resulting oligosaccharides could be broken down into monosaccharides, which eventually entered the glycolysis pathway to produce ATP or intermediates (e.g., pyruvate and alpha-ketoglutarate). These intermediates would go in different ways. For example, some intermediates can upregulate ketol-acid reductoisomerase (ilvC, Amuc_1178) and l-aspartate oxidase (nadB, Amuc_1079), while other intermediates may downregulate pyruvate dehydrogenase, dihydrodipicolinate reductase (dapB, Amuc_0257) and diaminopimelate dehydrogenase (ddh, Amuc_0581). Mucin addition upregulated succinyl-CoA ligase (sucC, Amuc_1713), but downregulated aconitate hydratase (acnA, Amuc_0904). These could reflect the regulations of ATP production.

In conclusion, mucin was found to be a good stimulus to proliferate this bacterium by upregulating expression of genes encoding glycosidase hydrolase, and cell shape and division. Mucin was also a good source of carbohydrates and amino acids for *A. muciniphila* to change its metabolic properties. High mucin addition upregulated genes involving nutrient uptake, cell growth, and cell morphology, probably by regulating ATP production. In a nutrient-enriched culture system, *A. muciniphila* showed a mucin-dependent growth by degrading mucin to maintain energy balance.

## Methods

### Bacterial strain and culture preparation

*A. muciniphila* (strain DSM 22959), which was stored in an anaerobic tube with 30% glycerol at – 80 °C, was obtained from DMSZ (German Collection of Microorganisms and Cell Culture)^46^. It was reactivated twice in BHI broth (Haibo, Qingdao, China, containing 10 g/L tryptone, 17.5 g/L beef heart powder, 2.0 g/L glucose, 5 g/L NaCl and 2.5 g/L Na_2_HPO_4_·12H_2_O), which was supplemented with 0.2% (m/v) porcine gastric mucin (Type III, Sigma-Aldrich, St, Louis, MO) for 48 h at 37 °C. Porcine gastric mucin (type III; Sigma-Aldrich, St. Louis, MO) was purified by ethanol precipitation as described previously^47^. Briefly, 10 g of mucin was stirred for 24 h at 4 °C in 500 mL of 0.02 M phosphate buffer (pH 7.4) containing 0.1 M NaCl and a few drops of toluene. After the first hour, the pH was adjusted to 7.2 with 2 M NaOH. After centrifugation at 10,000*g*, the supernatant was cooled to 0 °C and cooled ethanol (4 °C) was added to a final concentration of 60% (V/V). The resulting precipitate was suspended in 0.1 M NaCl and precipitated in 60% ethanol (V/V) at 4 °C. The total precipitate was washed with 100% ethanol and dissolved in distilled water. Then, the mucin solution was dialyzed against distilled water for 24 h at 4 °C. The dialyzed mucin solution was then lyophilized, dissolved in distilled water, and autoclaved before use. Then the optical density was measured under a microplate reader at 600 nm (Infinite M200, Tecan Group, Männedorf, Switzerland) and an inoculum with OD value of 0.15 was taken to prepare a solution of 10^8^ CFU/mL. Then the inoculum was incubated with 0% (control), 0.05%, 0.2% or 0.5% porcine gastric mucin (m/v, Sigma-Aldrich, St, Louis, MO) in a BHI medium (designated as BHI, BM0.05, BM0.2 and BM0.5, pH 7.0–7.4) respectively. There were three replicates each group at each time point, and all the samples were prepared with the same batch of mucin. All the medium solutions need to be preheated and an aliquot of 0.1% resazurin oxygen indicator (0.1 mL) was added to each medium. Then, 0.1 g l-cysteine hydrochloride was added under anaerobic condition and 9.5 mL medium was quickly dispensed into 30 mL serum bottles sealed with butyl-rubber stoppers. The medium was sterilized by autoclaving for 15 min at 121 °C. Incubations were performed in serum bottles sealed with butylrubber stoppers at 37 °C under anaerobic conditions provided by a gas phase of 182 kPa (1.8 atm) N_2_-CO_2_. The culture volume was 10 ml and the cultures were inoculated with 5 × 10^7^ bacterial cells from bacterial cultures. Negative controls comprised of a series of mucin media that was not inoculated. In addition, mucin medium was used to culture *A. muciniphila* according to the procedure of Derrien et al.^3^. The basic medium was prepared by dissolving 0.4 g KH_2_PO_4_, 0.76 g Na_2_HPO_4_·12H_2_O, 0.3 g NaCl, 0.3 g NH_4_Cl, 0.1 g MgCl_2_·6H_2_O, 0.11 g CaCl_2_, 4 g NaHCO_3_, 0.25 g Na_2_S_7_·9H_2_O, 0.5 mg resazurin and 5 g porcine gastric mucin in double distilled water, and mixing 1 mL acid trace element solution, 1 mL alkaline trace element solution and 1 mL vitamin solution, and finally making up to 1000 mL with double distilled water. Acid trace element solution was made up by dissolving 1.49 g FeCl_2_·4H_2_O, 0.06 g H_3_BO_3_, 0.07 g ZnCl_2_, 0.02 g CuCl_2_·2H_2_O, 0.06 g MnCl_2_, 0.12 g CoCl_2_·6H_2_O, and 0.02 g NiCl_2_·6H_2_O in double distilled water and 4.2 mL concentrated HCl was added and the final volume was 1000 mL. Alkaline trace element solution was prepared by dissolving 0.02 g Na_2_SeO_3_, 0.03 g Na_2_WO_4_·2H_2_O, 0.02 g Na_2_MoO_4_·2H_2_O and 0.4 g NaOH in 1000 mL double distilled water. Vitamin solution was prepared by dissolving 20 mg biotin, 200 mg niacin, 500 mg pyridoxine, 100 mg riboflavin, 200 mg thiamine, 100 mg cyanocobalamin, 100 mg p-aminobenzoic acid and 100 mg pantothenic acid in 1000 mL double distilled water. For solid plate culture, the normal saline for a dilution of bacterial culture needed to be preheated and 1% (V/V) of 0.1% resazurin oxygen indicator was added. Then, 1% (V/V) l-cysteine hydrochloride was added under anaerobic condition and 4.5 mL saline solution was quickly dispensed into 10 mL anaerobic tubes sealed with butyl-rubber stoppers. All the normal saline was sterilized by autoclaving for 15 min at 121 °C and 0.5 ml bacterial culture was injected into an anaerobic tube for serial dilution. Selected the appropriate dilution of bacterium for cultivation and counted at 37 °C in an anaerobic workstation (Bugbox M, Ruskinn Technology, Wales, UK).

### Scanning and transmission electron microscopy

Scanning electron microscopy was performed to visualize the shape of *A. muciniphila* as described by Bautista-Rosales et al.^48^. Images were obtained with a Hitachi S-3000N (Hitachi, Chiyoda, Tokyo, Japan). Transmission electron microscopy was applied to visualize ultrastructural changes in the microbial cells, the method was followed by Marrie et al.^49^. Finally, microbial structures were observed under a Hitachi h-7650 transmission electron microscope (Hitachi, Chiyoda, Tokyo, Japan).

### RNA isolation and analysis

A typical growth curve of a bacterium comprises of lag phase, log or exponential phase, stationary phase, death phase and/or prolonged stationary phase. The time from the early log phase to the early stationary phase, and the abundance and metabolites of a bacterium at the early stationary phase may reflect the growth performance of the bacterium in a specific medium^50^. Thus, RNA was extracted from the cultures at the time points of the maximal abundance of the bacterium (early stationary phase), i.e., 35 h for BHI, 31 h for BM0.05, 30 h for BM0.2, and 28 h for BM0.5. RNA extraction were treated with the TRIzol reagent (Thermo Fisher Scientific, Carlsbad, CA) according to the manufacturer’s protocols. The RNA quality was checked by 1% agarose gel electrophoresis, and the quantity and integrity were measured using a NanoDrop ND-2000 spectrophotometer (Thermo Fisher Scientific, Carlsbad, CA) and an Agilent 2100 Bioanalyzer (Agilent Technologies, Santa Clara, CA). Only samples with a RIN (RNA integrity number) score greater than 6.0 were used for library construction^51^.

A total amount of 3 μg RNA per sample was taken. Ribosomal RNA was removed using a Ribo-Zero rRNA removal kit (Illumina, San Diego, CA). First strand cDNA was synthesized using random hexamer primer and M-MuLV Reverse Transcriptase (RNaseH-). Second strand cDNA synthesis was subsequently performed using DNA Polymerase I and RNase H. In the reaction buffer, dNTPs with dTTP were replaced by dUTP. Remaining overhangs were converted into blunt ends via exonuclease/polymerase activities. After adenylation of 3′ ends of DNA fragments, NEBNext Adaptor with hairpin loop structure was ligated to prepare for hybridization. In order to select cDNA fragments of preferentially 150–200 bp in length, the library fragments were purified with AMPure XP system (Beckman Coulter, Beverly, MA). Then 3 μL USER Enzyme (New England Biolabs, Ipswich, MA) was used with size-selected, adaptor-ligated cDNA at 37 °C for 15 min followed by 5 min at 95 °C. Then PCR was done with Phusion High-Fidelity DNA polymerase (New England Biolabs, Ipswich, MA). Finally, products were purified (AMPure XP system) and library quality was assessed on an Agilent Bioanalyzer 2100 system (Agilent, Santa Clara, CA). A reference transcriptome was generated using *A. muciniphila* under BHI medium. Twelve libraries were constructed from the four different groups, and raw reads were obtained from HiSeq 2500 sequencing system (Illumina, San Diego, CA). Clean data were obtained by removing reads containing adapter and ploy-N, and low quality reads from raw data. At the same time, Q20, Q30 and GC content in the clean data were calculated. All the downstream analyses were based on clean data with high quality.

Clean data was transformed by log10 arithmetic and PCA was performed on the transformed data using DESeq2 (R package, version 1.30.0). In addition, the same R package was applied to acquire Venn diagrams to reflect the number of DEGs and heatmap to visualize the gene expression patterns. GO enrichment analysis of DEGs was performed by the GOseq R package (version 1.30.0, https://www.r-project.org/) and KOBAS (version 2.0, http://kobas.cbi.pku.edu.cn/) was applied to test the significant enrichment of DEGs in KEGG pathways. GO terms and KEGG pathways with *P* values smaller than 0.05 were considered significantly enriched. Raw sequencing data can be accessed through SRA accession no. PRJNA559704. The genome sequence of *A. muciniphila* was deposited in GenBank under the accession number CP042830.

### Quantitative RT-PCR analysis

Several genes involving mucin degradation were selected for quantitative reverse transcription-PCR (qRT-PCR) to validate if these enzymes were highly expressed, and each group had three replicates. Total RNA was isolated as described above and the cDNA was synthesized using the PrimeScript RT Master Mix kit (Takara, Kusatsu, Shiga, Japan) following the manufacturer^’^s protocol. qRT-PCR was carried out using the SYBR Green probe on a QuantStudio 6 Flex system (Applied Biosystems, Foster City, CA). The cycling conditions were set as follows: initial denaturation at 95 °C for 30 s, and then 40 amplification cycles of 95 °C for 5 s and 60 for 34 s. Melting curve analysis was used to validate the specificity of primers. Levels of target gene transcripts were calculated relative to the 16S rRNA using the 2^−△△Ct^ method to normalize expression levels^3^. Primers are listed in supplemental materials Table S11.

### Determination of extracellular glycoside hydrolase activity

The activities of β-hexosaminidase, β-galactosidase and α-l-fucosidase were determined as described by Wendeler and Elhenawy et al.^52,53^. Briefly, 10 mL of bacterial cultures at the time points of the maximal abundance of the bacterium were centrifuged at 10,000×*g* for 10 min and the supernatant was passed through a 0.22-μm Millipore membrane. Twenty microliters of the filtrate was incubated with 80 µL 50 mM phosphate buffer solution (PBS, pH7.0). To determine the activities of β-hexosaminidase, β-galactosidase and α-l-fucosidase, appropriate amount of 4-nitrophenyl-N-acetyl-β-d-glucosaminide, 4-nitrophenyl-β-d-galactopyranoside or 2-chloro-4-nitrophenyl-α-l-fucopyranoside was added, respectively, as substrates to a final concentration of 1 mmol/L. The β-hexosaminidase and β-galactosidase involving reactions were done at 37 °C for 1 h. The α-l-fucosidase involving reaction was done at 37 °C for 2.5 h. All these enzymatic reactions were stopped by adding 100 μL 1 M Na_2_CO_3_ and the absorbance was read at 405 nm. Each group had three replicates.

### Metabolome profiling by gas chromatography-mass spectrometry (GC–MS)

The cultures were taken at the time points of the maximal abundance of the bacterium, i.e., 35 h for BHI, 31 h for BM0.05, 30 h for BM0.2, and 28 h for BM0.5 (n = 5 each) for non-targeted metabolomic analysis as described in Mourão et al.^54^. GC–MS analysis was performed by Moros et al.^55^. Metabolites were identified by analysis of variance using the SAS software (version 8.0.1, Cary, NC). Significant differences were declared at the level of 0.05. KEGG and functional enrichment analyses were performed via omicsbean (http://www.omicsbean.cn/). Orthogonal partial least multiplication discriminant analysis (OPLS-DA) was done under the SIMCA 14.1 program. The data of metabolome was deposited in iProX under the project id PXD024558.

### Determination of glucose and fructose

To validate the ability of *A. muciniphila* to utilize monosaccharides, glucose and fructose concentrations in the culture were monitored during the growth of bacteria at five time points (0 h, 9 h, 18 h, 27 h and 36 h). Five milliliters of culture were ultrasonically treated with 200 W for 3 s with a burst of 10 s. The treatment was repeated 100 times. The resulting mixture was heated in a 95 °C water bath for 10 min. The mixture was centrifuged at 1000×*g* at 4 °C for 10 min. Glucose and fructose in the supernatant were quantified by commercial kits (No. F006 and No. A085, Jiancheng, Nanjing, China).

### Statistical analysis

All the data were checked for normal distribution. The effect of mucin addition on the measured variables, culture absorbance, PCR data of 16 genes of interest, enzymatic activities, glucose and fructose concentrations, and amino acids was evaluated by one-way ANOVA and comparisons were performed by t test using the SAS software (version 8.0.1, Cary, NC). Significant differences were declared if *P* value was less than 0.05.

## Supplementary Information

Supplementary Information.
