# Supplementary material for: Transcriptomics and metabolomics reveal the adaption of Akkermansia muciniphila to high mucin by regulating energy homeostasis
Source: Sci Rep. 2021 Apr 27;11:9073. doi: 10.1038/s41598-021-88397-z (PMC8079684; doi:10.1038/s41598-021-88397-z)
Supplement: Supplementary file 1 — Supplementary Information. [file 41598_2021_88397_MOESM1_ESM.docx]

**Transcriptomics and metabolomics reveal the adaption of *Akkermansia muciniphila* to high mucin by regulating energy homeostasis**

Xinyue Liu^1^, Fan Zhao^1^, Hui Liu^1^, Yunting Xie^1^, Di Zhao^1^,Chunbao Li^1,2^*

^1^ Key Laboratory of Meat Processing and Quality Control, Ministry of Education; Key Laboratory of Meat Processing, Ministry of Agriculture and Rural Affairs; Jiangsu Collaborative Innovation Centre of Meat Production and Processing, Quality and Safety Control, Meat Production; College of Food Science and Technology, Nanjing Agricultural University; Nanjing 210095, P.R. China

^2^ National Center for International Research on Animal Gut Nutrition, Nanjing Agricultural University; Nanjing 210095, P.R. China

*Correspondence:

Dr. Chunbao Li

Nanjing Agricultural University, Weigang 1#, Nanjing, 210095, China

E-mail: [chunbao.li@njau.edu.cn](mailto:chunbao.li@njau.edu.cn)

Tel/Fax: 86 25 84395679

**General genomic information of *A.muciniphila***

The complete genome of DSM 22959 consisted of a single circular chromosome of 2664043 bp and contained 2568 predicted genes, 2213 protein coding genes, 53 tRNA genes, 9 rRNA genes and 55.76%GC content. Genome features of *A. muciniphila* were summarized in Fig S1 and Table S1. Functional annotations of the predicted genes were listed in Table S2. In the COG database, 180, 178, 153 and 129 genes were annotated to “cell wall, membrane, envelope biogenesis”, “translation, ribosomal structure and biogenesis”, “amino acid transport and metabolism” and “carbohydrate transport and metabolism” (Fig. S2A). In the GO database, catalytic activity, cell part and cell and metabolic process were annotated (Fig. S2B). In the KEGG database, metabolic functions of carbohydrate, amino acid, cofactors and vitamins were annotated (Fig. S2C). Venn diagram indicated that these five strains of *A. muciniphila* had a great homogeneity in their genome sequences and 1708 consensus gene sets were generated (Fig. S2D). The studied strain, ATCC_BAA835, YL44, AMDK_1 and AMDK_3 had 325, 25, 167, 251 and 129 unique gene sets，respectively. The unique genes in the studied strain mainly encode proteins involving DNA, RNA, translation, membrane, ATPase, sulfuric ester hydrolase, beta-galactosidase and oxidoreductase.

**Genome sequencing, assembly and gene annotation**

DNA alteration may occur during microbial transmission for many generations due to changes in the medium composition and environmental temperature. And thus we performed whole-genome sequencing of *A. muciniphila* was to establish a set of reference sequences for transcriptome analysis. The strain of *A. muciniphila* (strain DSM 22959) was reactivated. The DNA was extracted using a bacterial genomic DNA extraction kit (Takara, Kusatsu, Shiga, Japan) from 10 mL of bacterial culture. The quality of DNA samples was tested by agarose gel electrophoresis and quantified by a Qubit fluorometer (Life Technologies, Carlsbad, CA).The extracted genomic DNA was used to construct a 10 kb SMRTbell library. The genome of *A. muciniphila* was sequenced by single molecule, real-time (SMRT) technology. Low-quality reads were filtered out by the SMRT algorithm (version 2.3.0, PacBio, Menlo Park, CA)^1^and the reads were assembled to generate the complete genome by SOAP denovo (version 2.0, https://sourceforge.net/projects/soapdenovo2/).

GeneMarks (version 4.17, http://topaz.gatech.edu/) was used for gene prediction to retrieve the related coding genes^2^. Transfer RNAs (tRNAs) were predicted with tRNAscan-SE (version 1.3.1), and ribosome RNAs (rRNAs) were predicted with rRNAmmer (version 1.2), and finally sRNAs were predicted by BLAST against Rfam database using Rfam sofeware (version 12.2)^3,4,5^. A whole-genome blast search (E-value less than 10^-5^, minimal alignment length percentage larger than 40%) was performed against the Gene Ontology (GO), Kyoto Encyclopedia of Genes and Genomes (KEGG) and Clusters of Orthologous Groups (COG) databases, NR database (Non-Redundant Protein Database) and Swiss-Prot database for gene annotation. The genome sequence of *A. muciniphila* had been deposited GenBank under the accession number CP042830.Core genes and specific genes were analyzed by the Cluster Database at High Identity with Tolerance (CD-HIT, version 4.6.1, http://www.bioinformatics.org/cd-hit/) with a threshold of 50% pairwise identity and 0.7 length difference cut-off on the amino acid level. Venn diagrams were generated to show the relationships among the samples using the R language (version 1.30.0, https://www.r-project.org). Other four strains of whole genome information were downloaded in NCBI database.


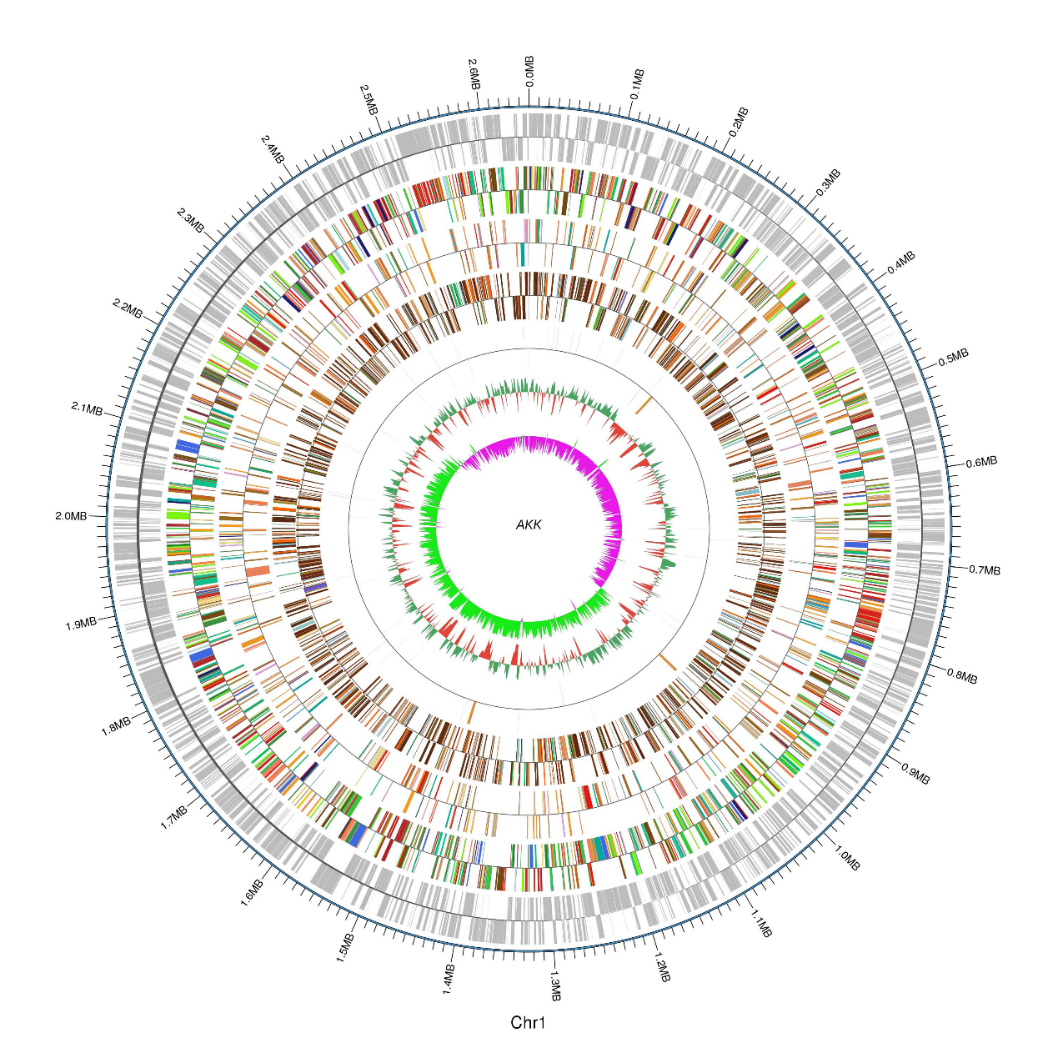


**Fig S1** A whole-genome mapping of the studied strain DSM 22959. The outermost circle is the genomic sequence coordinate. The outer to center circles show (i) ending genes (gray); (ii) the annotations of encoding genes in the COG database; (iii) the annotations of encoding genes in the KEGG database; (iv) the annotations of encoding genes in the GO database; (v) the annotations of encoding genes in the ncRNA database; and (vi) the distribution of GC% and GC skew (G-C)/(CG). The red part indicates that the GC content in this region is lower than the whole genome average GC content, while the green part is the opposite. The higher the peak value, the greater difference existed from the average GC content. The fuchsia part indicates that the content of G in the region is lower than the content of C. The grass-green part is the opposite.


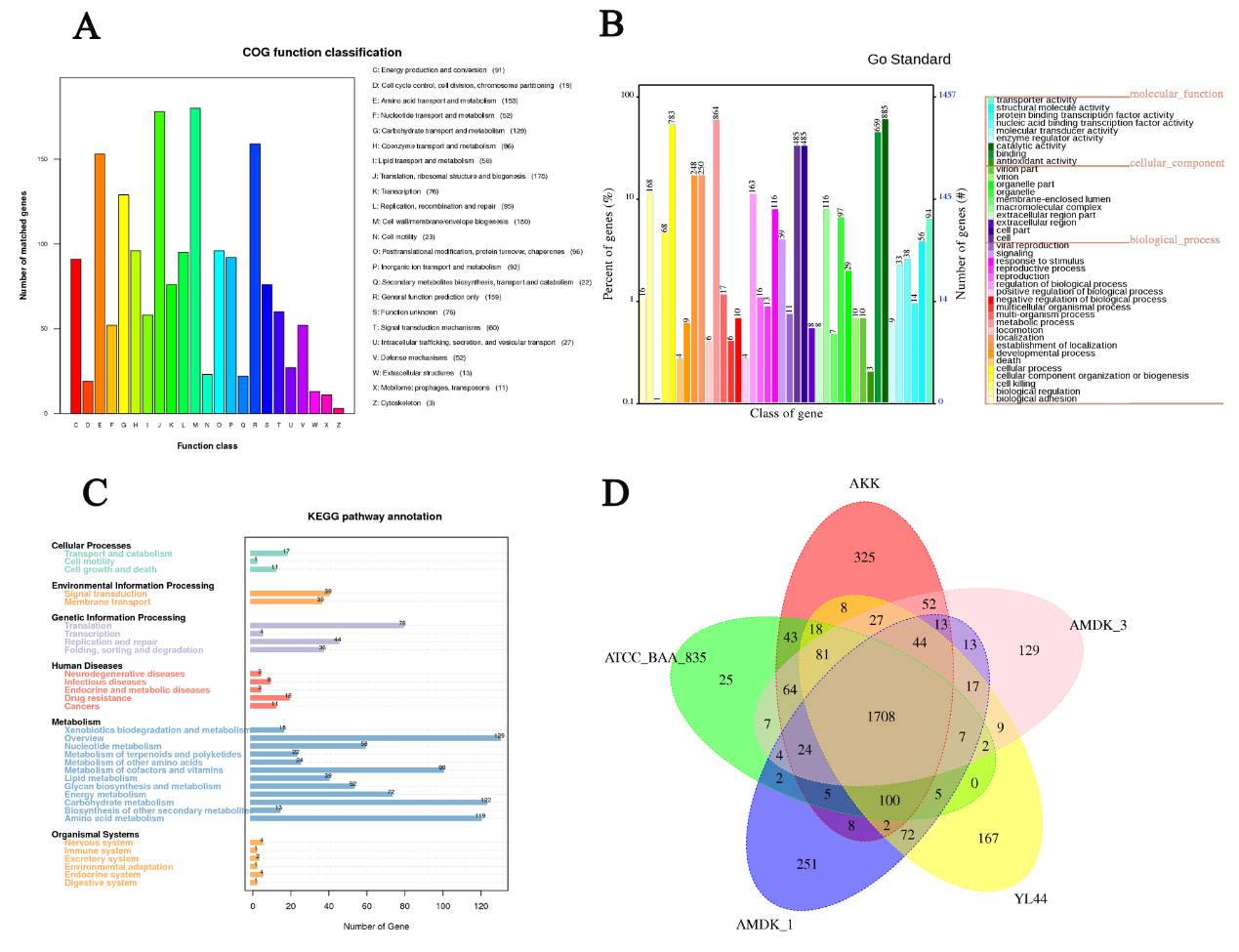


**Fig S2** Gene annotations of the encoded genes in databases of COG (A), GO (B), and KEGG (C). (D) Venn diagram of genes in different strains of *A. muciniphila* showing the number of consensus or unique gene sets. Generally, the protein sequence encoded by genes greater than 50%, and the difference of sequence length less than 0.3 is regarded as a consensus gene set.

| Table S1 Summary of genome feature of *A.muciniphila* | |
| --- | --- |
| Genome | Value |
| Number of Reads | 108324 |
| Number of Bases，bp | 1278150994 |
| Mean Read Length，bp | 11799 |
| N50 Read Length，bp | 16244 |
| Mean Read Score | 0.84 |
| Chromosome Size,bp | 2664043 |
| Max Contig Length，bp | 2,677,349 |
| N50 Contig Length，bp | 2,677,349 |
| Sum of Contig Lengths，bp | 2,677,349 |
| GC content (%) | 56.64 |
| No. of protein-coding genes | 2568 |

| Table S2 Summary of functional annotation of the predicted genes of *A. muciniphila* | |
| --- | --- |
| Abbreviation: Nr, Non-redundant database; Clusters of Orthologous Groups(COG); Gene ontology (GO); KEGG, Kyoto Encyclopedia of Genes and Genomes. | |
| Type | Numbers |
| Nr | 2,197 |
| COG | 1602 |
| GO | 1456 |
| KEGG | 1,095 |
| Swiss-Prot | 676 |

References:

1.Berlin, K. *et al*. Assembling large genomes with single-molecule sequencing and locality sensitive hashing. *Nature Biotechnology* **33**, 623-630(2015).

2.Besemer, J., Lomsadze, A. & Borodovsky, M. GeneMarkS: a self-training method for prediction of gene starts in microbial genomes. Implications for finding sequence motifs in regulatory regions. *Nucleic Acids Research* **29**, 2607-2618(2001).

3. Lowe, T.M. & Eddy, S.R. tRNAscan-SE: a program for improved detection of transfer RNA genes in genomic sequence. *Nucleic Acids Research* **25**, 0955–964(1997).

4. Lagesen, K. *et al*. RNAmmer: consistent and rapid annotation of ribosomal RNA genes. *Nucleic Acids Research* **35**, 3100–3108(2007).

5. Gardner, P. P. *et al*. Rfam: updates to the RNA families database. *Nucleic Acids Research* **37**, D136–D140(2009).


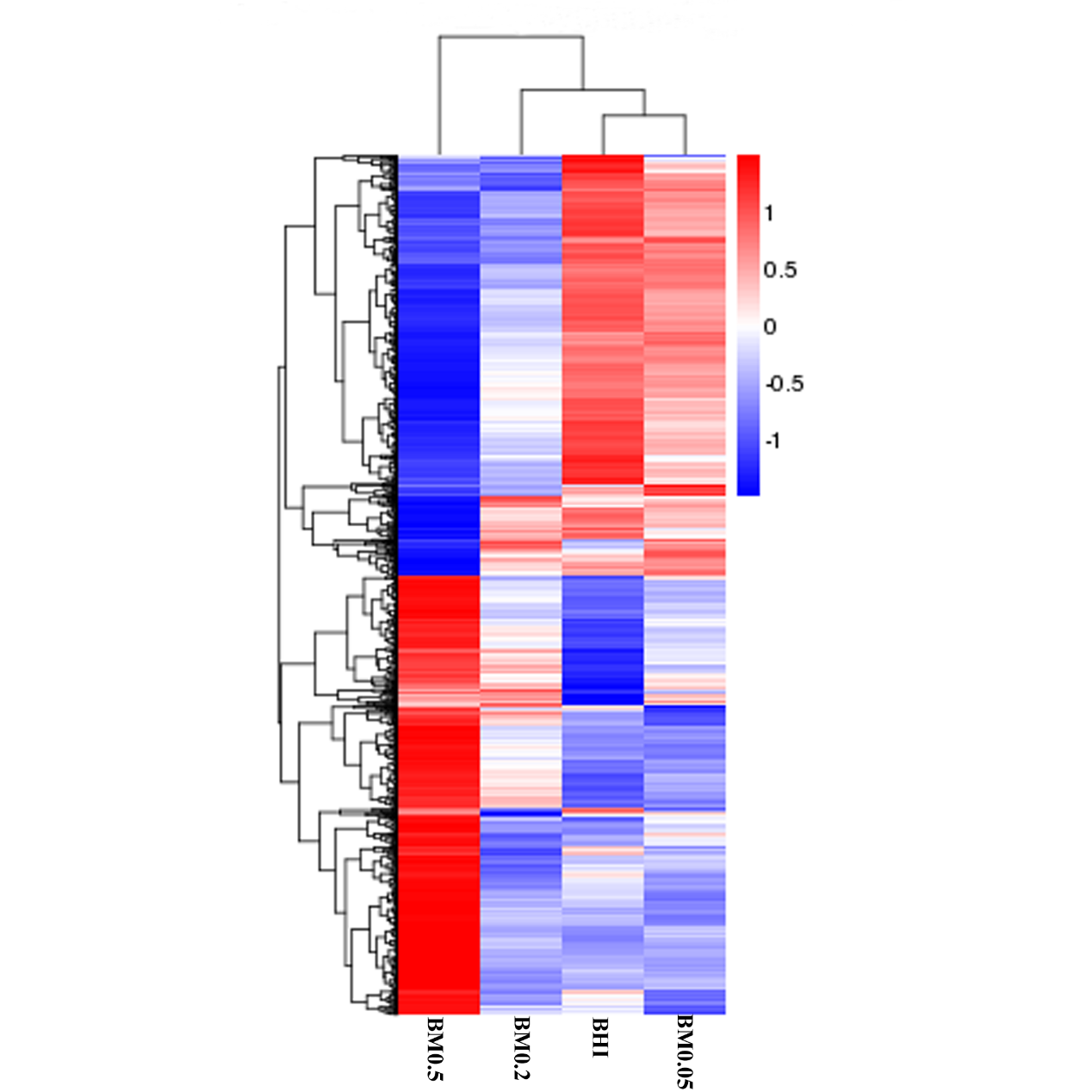


**Figure S3** Clustering analysis of DEGs in the four groups. Notes: Red indicates a higher expression pattern, and blue indicates a lower expression pattern. The color from red to blue indicates log10 (FPKM+1) from high to low values.


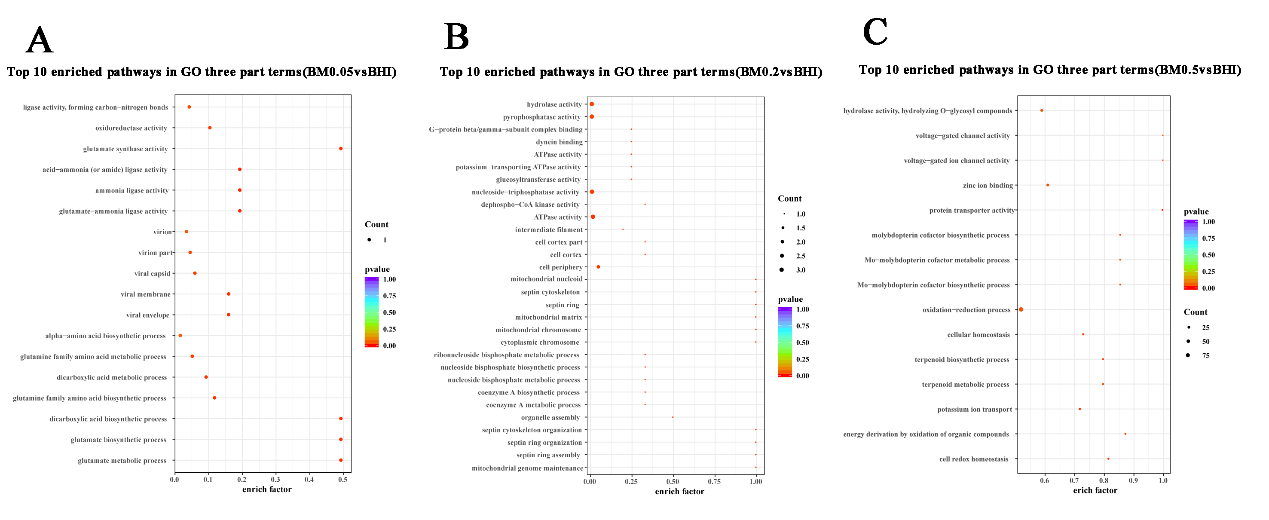


**Figure S4** Significantly enriched GO pathways of DEGs **(A)** to **(C)** Top 10 most enriched GO three part terms in BM0.05, BM0.2 and BM0.5 groups, respectively. Pval less than 0.05 was regarded as a significantly enriched pathway.


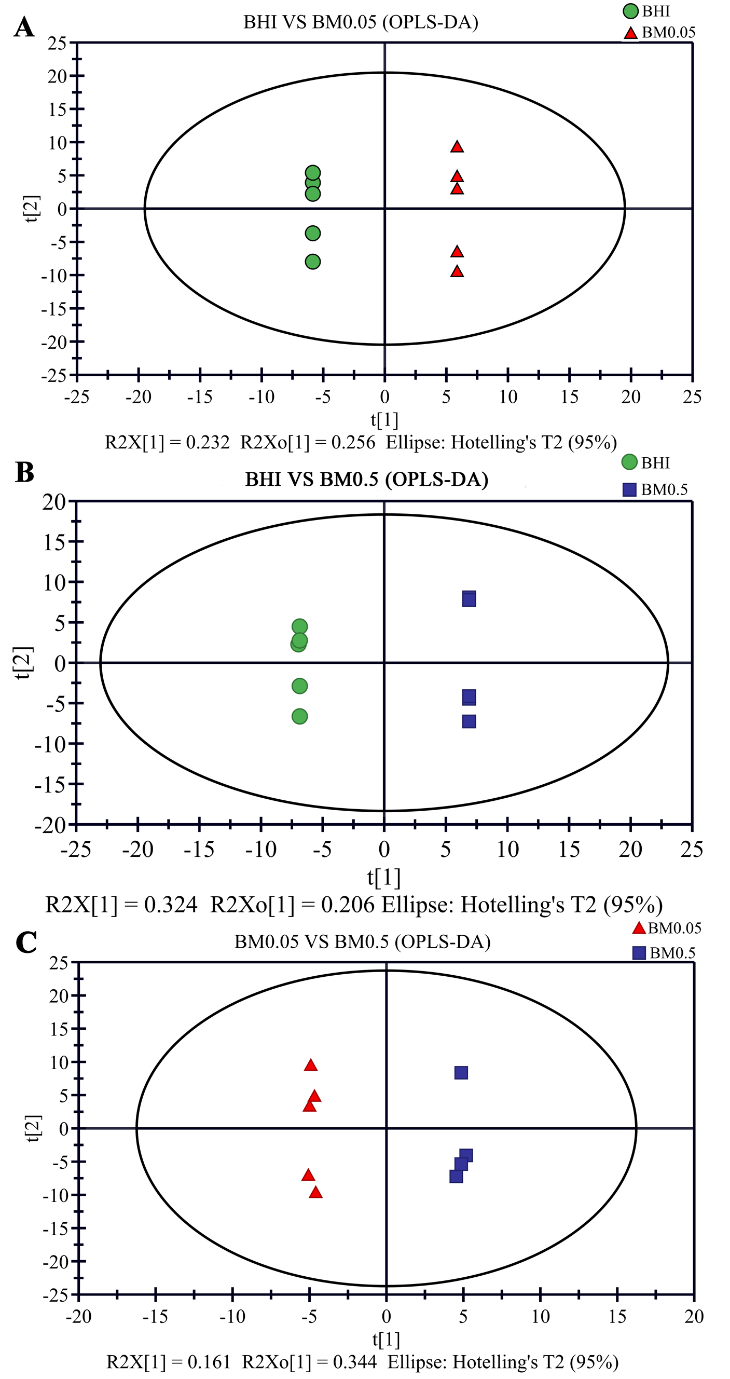


**Figure S5** OPLS analysis of metabolomics data. (A) BM0.05 vs. BHI groups; (B) BM0.5 vs. BHI groups; (C) BM0.5 vs BM0.05 groups.

Table S3 Summary of *RNA-seq* data statistics

| Sample name | Raw reads(10^7^) | Clean reads(10^7^) | clean bases | Error rate(%) | Q20(%) | Q30(%) | GC content(%) | rRNA(%) |
| --- | --- | --- | --- | --- | --- | --- | --- | --- |
| BHI_1 | 2.12 | 2.04 | 3.06G | 0.01 | 97.9 | 94.10 | 58.02 | 0.10 |
| BHI_2 | 2.09 | 2.01 | 3.01G | 0.02 | 97.4 | 92.70 | 57.65 | 0.31 |
| BHI_3 | 1.50 | 1.47 | 2.20G | 0.02 | 95.7 | 89.11 | 58.14 | 0.09 |
| BM0.05_1 | 1.53 | 1.49 | 2.23G | 0.02 | 96.1 | 89.93 | 57.50 | 0.16 |
| BM0.05_2 | 2.06 | 1.98 | 2.97G | 0.01 | 97.8 | 93.78 | 57.23 | 0.09 |
| BM0.05_3 | 2.13 | 2.05 | 3.08G | 0.02 | 97.6 | 93.25 | 57.57 | 0.15 |
| BM0.2_1 | 1.85 | 1.78 | 2.68G | 0.01 | 98.0 | 94.15 | 56.40 | 0.15 |
| BM0.2_2 | 2.07 | 1.99 | 2.99G | 0.02 | 97.4 | 92.76 | 56.30 | 0.15 |
| BM0.2_3 | 3.33 | 3.23 | 4.84G | 0.01 | 98.6 | 95.83 | 56.46 | 0.04 |
| BM0.5_1 | 2.06 | 1.98 | 2.97G | 0.02 | 96.8 | 91.88 | 51.09 | 0.26 |
| BM0.5_2 | 1.98 | 1.90 | 2.86G | 0.02 | 97.1 | 92.46 | 52.29 | 0.29 |
| BM0.5_3 | 2.13 | 2.05 | 3.08G | 0.02 | 97.1 | 92.37 | 51.69 | 0.35 |

Table S4The numbers of DEGs significantly enriched in KEGG pathways

|  | Pathway | P-value | Number of DEGs | |
| --- | --- | --- | --- | --- |
|  |  |  | upregulated | downregulated |
| BM0.05 vs BHI | Alanine, aspartate and glutamate metabolism | 0.008 | 2 |  |
| BM0.2 vs BHI | Starch and sucrose metabolism | 0.025 | 1 |  |
|  | Two-component system | 0.042 | 1 |  |
| BM0.5 vs BHI | Other glycan degradation | 5.90E-05 | 12 |  |
|  | Ribosome | 0.011 | 17 |  |
|  | Sulfur metabolism | 0.014 | 5 |  |
|  | Two-component system | 0.032 | 8 |  |
| BM0.5 vs BM0.05 | Ribosome | 0.001 | 21 |  |
|  | Other glycan degradation | 0.001 | 10 |  |
|  | Sulfur metabolism | 0.016 | 5 |  |
| BM0.5 vs BM0.2 | Sulfur metabolism | 0.0002 | 4 |  |
|  | Other glycan degradation | 0.002 | 4 |  |
|  | Biotin metabolism | 0.043 |  | 2 |

Table S5 DEGs related to replication, recombination and repair

| Gene ID | Symbol | Log2 FC | P_adj_ | Description |
| --- | --- | --- | --- | --- |
| AKKGM000447 | dnaE | 0.55 | 0.048 | DNA polymerase III subunit alpha |
| AKKGM000716 | holB | -0.54 | 0.049 | DNA polymerase III subunit delta |
| AKKGM001445 | dnaX | 1.50 | 5.93E-09 | DNA polymerase III subunit gamma/tau |
| AKKGM001446 | dnaX | 5.25 | 1.40E-12 | DNA polymerase III subunit gamma/tau |
| AKKGM001606 | dnaB | 0.98 | 0.040 | replicative DNA helicase |

Table S6 The summary of the R2X and Q2 value of the O-PLS models

|  | BM0.5vsBHI | BM0.05vsBHI | BM0.5vsBM0.05 |
| --- | --- | --- | --- |
| R2X | 0.86 | 0.81 | 0.59 |
| Q2（cum） | 0.96 | 0.75 | 0.81 |

Table S7 Summary of variance analysis results for being identified compounds

| Metabolites | BHI | BM0.05 | BM0.5 | P values |
| --- | --- | --- | --- | --- |
| Sorbose | 28.67±7.02a | 15.12±2.02b | 6.32±3.05c | <0.0001 |
| Mannose | 45.49±10.07a | 34.85±6.43b | 1.31±0.71c | <0.0001 |
| 2,7-anhydro-beta-Sedoheptulose | 2.88±0.56a | 1.66±0.28b | 0.60±0.16c | <0.0001 |
| Fructose | 15.68±2.96a | 10.36±2.47b | 3.79±3.15c | 0.0001 |
| similar to Glycerolaldopyranosid | 0.59±0.18a | 0.27±0.11b | 0.11±0.03b | 0.0002 |
| Valine | 0.27±0.05b | 0.23±0.03b | 0.43±0.08a | 0.0004 |
| Lactic acid dimer | 0.94±0.14a | 0.56±0.11b | 0.55±0.14b | 0.0008 |
| Lysine | 59.20±6.59a | 42.76±8.15b | 37.94±5.50b | 0.0009 |
| Galactinol | 0.73±0.08a | 0.58±0.11b | 0.46±0.06c | 0.001 |
| Spermidine | 1.91±0.28a | 1.19±0.31b | 1.15±0.23b | 0.001 |
| Hexanoic acid | 0.12±0.04b | 0.12±0.02b | 0.36±0.15a | 0.002 |
| Glucopyranose | 33.10±11.96a | 24.78±13.51a | 3.25±2.41b | 0.002 |
| Hydrocaffeic acid | 2.57±0.30a | 1.81±0.36b | 1.68±0.33b | 0.002 |
| Leucine | 3.24±0.82b | 2.83±0.53b | 5.36±1.32a | 0.003 |
| 3-Hydroxytetradecanoic_acid | 15.31±2.49a | 14.35±2.19a | 7.48±4.08b | 0.003 |
| Xylose | 0.36±0.07a | 0.21±0.01b | 0.23±0.07b | 0.003 |
| Erythrose | 3.20±0.46a | 2.36±0.35b | 1.96±0.57b | 0.004 |
| Galactose | 8.02±2.12a | 5.36±1.45b | 3.99±0.57b | 0.004 |
| Alanyl-alanine | 8.61±1.55a | 6.28±1.29b | 5.38±0.82b | 0.005 |
| beta-Alanine | 2.19±0.23a | 1.67±0.32b | 1.44±0.32b | 0.005 |
| Glycolic acid | 0.61±0.14b | 0.49±0.13b | 0.93±0.24a | 0.005 |
| Asparagine | 21.21±2.70a | 16.02±3.38b | 14.73±2.14b | 0.007 |
| Dodecanol | 0.43±0.07a | 0.27±0.11b | 0.24±0.07b | 0.009 |
| 2-hydroxy-Butanoic acid | 2.27±0.34a | 1.70±0.27b | 1.61±0.32b | 0.012 |
| Phenylalanine | 306.53±39.31a | 207.16±64.91b | 187.34±61.64b | 0.013 |
| Ornithine | 37.33±4.43a | 28.91±6.12b | 27.16±3.62b | 0.013 |
| 3-iodo-Tyrosine | 14.28±4.79a | 9.36±1.92b | 6.89±3.03b | 0.016 |
| N-methyl- Glutamic acid | 0.56±0.09b | 0.49±0.10b | 0.73±0.14a | 0.017 |
| 5-hydroxy-Lysine | 109.61±17.50a | 93.38±11.71ab | 71.58±22.39b | 0.017 |
| N-acetyl- Phenylalanine | 0.87±0.39b | 1.08±0.28b | 1.68±0.48a | 0.018 |
| 5-hydroxy-Tryptamine | 3.68±0.46a | 2.73±0.72b | 2.43±0.62b | 0.018 |
| 2-amino-Butanoic acid | 10.12±1.73a | 7.34±1.62b | 6.63±1.88b | 0.019 |
| 6-deoxy-Mannopyranose | 3.24±1.11a | 2.08±0.33b | 1.81±0.45b | 0.019 |
| Purine | 14.55±3.90a | 9.58±1.58b | 9.63±2.08b | 0.020 |
| 2-amino-2-deoxy-D-Galactose | 0.38±0.21a | 0.14±0.14b | 0.10±0.06b | 0.020 |
| N-acetyl-Neuraminic acid | 0.02±0.02b | 0.02±0.01b | 0.33±0.29a | 0.021 |
| Citric acid | 15.80±2.73a | 12.42±0.92b | 13.08±1.28b | 0.029 |
| Xylitol | 0.15±0.06b | 0.10±0.03b | 0.62±0.51a | 0.032 |
| 5-methyl-Hydantoin | 0.93±0.092a | 0.68±0.15b | 0.75±0.14ab | 0.035 |
| Diisopropanolamine | 1.33±0.45a | 0.91±0.42ab | 0.57±0.34b | 0.036 |
| Glutamic acid | 3.49±0.69a | 2.67±0.32b | 2.67±0.42b | 0.037 |
| Inosine | 2.81±0.77a | 2.19±0.37ab | 1.88±0.20b | 0.037 |
| Threonine | 65.60±9.47a | 50.61±11.62b | 48.19±9.51b | 0.041 |
| 4-amino-3-hydroxy-Butanoic acid | 2.34±0.27a | 1.85±0.29b | 1.86±0.36b | 0.041 |
| N-acetyl-Methionine | 0.04±0.02b | 0.17±0.28ab | 0.74±0.66a | 0.046 |
| Dihydroxyphenylalanine | 1.47±0.34a | 1.06±0.29ab | 0.85±0.41b | 0.047 |
| Adenine | 5.00±0.89ab | 4.02±0.87b | 5.90±1.33a | 0.047 |
| N-acetyl-5-hydroxy-Tryptamine | 0.31±0.09ab | 0.25±0.07b | 0.54±0.27a | 0.047 |
| 5-hydroxy-1H-Indole-3-acetic acid | 0.23±0.07a | 0.10±0.11b | 0.12±0.05ab | 0.049 |

a,b Means with different letters differ significantly (P<0.05). Items in red color increased with levels of mucin addition. Items in blue color decreased with levels of mucin addition.

Table S8 DEGs related to threonine between BM0.5 and BHI groups

| Gene ID | Swiss Prot ID | Log2 FC | P_adj_ | Description |
| --- | --- | --- | --- | --- |
| AKKGM000105 | Q8KY50 | -0.71 | 0.0085 | Serine/threonine-protein kinase |
| AKKGM001664 | P54734 | -1.03 | 0.0001 | Serine/threonine-protein kinase |
| AKKGM001731 | A0LIN6 | -1.47 | 1.01E-07 | 4-hydroxythreonine-4-phosphate dehydrogenase |
| AKKGM002030 | P56881 | -0.66 | 0.0134 | Threonine--tRNA ligase |
| AKKGM002346 | A6QGC0 | -0.80 | 0.0054 | Serine/threonine-protein kinase |

Table S9 Annotations of DEGs associated with metabolites in response to mucin

| pathway：Amino sugar and nucleotide sugar metabolism | | | | |
| --- | --- | --- | --- | --- |
| Metabolites：Mannose、Fructose | | | | |
| Gene ID | Locus tag | Log2（BM0.5/BHI） | Log2（BM0.5/BM0.05） | Product or description |
| up |  |  |  |  |
| AKKGM000438 | Amuc_0369 | 2.07 | 2.04 | hexosaminidase |
| AKKGM001027 | Amuc_0868 | 2.25 | 1.82 | hexosaminidase |
| AKKGM001955 | Amuc_1669 | 2.06 | 2.07 | hexosaminidase |
| AKKGM002135 | Amuc_1822 | 1.51 | 1.53 | glucosamine-6-phosphate deaminase |
| AKKGM002285 | Amuc_1946 | 0.85 | 0.56 | N-acetylneuraminate lyase |
| AKKGM002370 | Amuc_2018 | 1.71 | 1.50 | hexosaminidase |
| AKKGM002515 | Amuc_2136 | 0.74 | NA | hexosaminidase |
| AKKGM002529 | Amuc_2148 | NA | 0.69 | beta-N-acetylhexosaminidase |
| down |  |  |  |  |
| AKKGM000096 | Amuc_0075 | -1.27 | -0.78 | fructokinase |
| AKKGM000124 | Amuc_0097 | -0.97 | -0.70 | glucokinase |
| AKKGM000190 | Amuc_0155 | -0.84 | -0.76 | phosphomannomutase |
| AKKGM001145 | Amuc_0969 | NA | -0.69 | galactokinase |
| AKKGM002040 | Amuc_1741 | -0.78 | -0.70 | UTP--glucose-1-phosphate uridylyltransferase |
| AKKGM002252 | Amuc_1919 | -1.13 | -0.71 | mannose-1-phosphate guanylyltransferase |
| AKKGM002328 | Amuc_1975 | -1.31 | -1.23 | glucose-6-phosphate isomerase |
| pathway：Galactose metabolism | | | | |
| Mannose、Galactinol | | | | |
| up |  |  |  |  |
| AKKGM000347 | Amuc_0290 | 0.90 | 0.67 | beta-galactosidase |
| AKKGM001401 | Amuc_1187 | 1.31 | 1.03 | alpha-galactosidase |
| AKKGM001951 | Amuc_1666 | 2.34 | NA | beta-galactosidase |
| AKKGM001950 | bhl:Bache_0093 | 2.62 | 2.05 | beta-galactosidase |
| down |  |  |  |  |
| AKKGM000124 | Amuc_0097 | -0.97 | -0.70 | glucokinase |
| AKKGM000190 | Amuc_0155 | -0.84 | -0.76 | phosphomannomutase |
| AKKGM001145 | Amuc_0969 | NA | -0.69 | galactokinase |
| AKKGM001735 | Amuc_1481 | NA | -0.64 | 6-phosphofructokinase |
| AKKGM002040 | Amuc_1741 | -0.78 | -0.70 | UTP--glucose-1-phosphate uridylyltransferase |
| AKKGM002193 | Amuc_1870 | -0.86 | -0.77 | alpha-glucosidase |
| pathway：ABC transporter | | | | |
| Metabolites：Mannose、Spermidine、Putrescine、Xylitol | | | | |
| up |  |  |  |  |
| AKKGM001392 | Amuc_1179 | 2.08 | 1.33 | iron complex transport system ATP-binding protein |
| AKKGM001527 | Amuc_1303 | 1.23 | 1.47 | phosphate transport system ATP-binding protein |
| AKKGM001530 | Amuc_1306 | NA | 1.08 | phosphate transport system substrate-binding protein |
| AKKGM001531 | Amuc_1307 | NA | 1.48 | phosphate transport system ATP-binding protein |
| AKKGM002262 | Amuc_1928 | 1.27 | 1.21 | iron complex transport system substrate-binding protein |
| AKKGM002175 | Amuc_1853 | 0.65 | 0.59 | peptide/nickel transport system substrate-binding protein |
| down |  |  |  |  |
| AKKGM000176 | Amuc_0143 | -1.38 | -1.02 | peptide/nickel transport system permease protein |
| AKKGM000177 | Amuc_0144 | -1.65 | -1.50 | peptide/nickel transport system permease protein |
| AKKGM000178 | Amuc_0145 | -0.64 | NA | peptide/nickel transport system substrate-binding protein |
| AKKGM000191 | Amuc_0156 | -1.45 | -1.29 | ATP-binding cassette, subfamily B, bacterial MsbA |
| AKKGM000195 | Amuc_0159 | -1.38 | -1.22 | putative ABC transport system permease protein |
| AKKGM000221 | Amuc_0182 | -0.63 | NA | lipoprotein-releasing system permease protein |
| AKKGM000381 | Amuc_0320 | -0.70 | -0.68 | peptide/nickel transport system ATP-binding protein |
| AKKGM001415 | Amuc_1199 | -2.73 | -2.56 | cobalt/nickel transport system permease protein |
| AKKGM001416 | Amuc_1200 | -1.43 | -1.07 | cobalt/nickel transport system permease protein |
| AKKGM002177 | Amuc_1855 | -0.75 | NA | peptide/nickel transport system permease protein |

Table S10 Summary of specific significantly enriched pathways of differentially expressed genes associated with metabolites of *A. muciniphila* in BM0.5

| pathway：Pyrimidine metabolism | |  |  |
| --- | --- | --- | --- |
| Metabolites：beta-Alanine、Thymine、Uracil | | |  |
| Gene ID | Locus tag | Log2（BM0.5/BHI） | Product or description |
| up |  |  |  |
| AKKGM001445 | Amuc_1228 | 1.50 | DNA polymerase III subunit gamma/tau |
| AKKGM000207 | Amuc_0170 | 1.23 | CTP synthase |
| AKKGM001469 | Amuc_1251 | 0.75 | carbamoyl-phosphate synthase large subunit |
| AKKGM001763 | Amuc_1505 | 0.74 | DNA-directed RNA polymerase subunit alpha |
| AKKGM001231 | Amuc_1041 | 0.69 | DNA-directed RNA polymerase subunit beta |
| AKKGM001545 | Amuc_1319 | 0.59 | uridylate kinase |
| AKKGM000447 | Amuc_0374 | 0.55 | DNA polymerase III subunit alpha |
| down |  |  |  |
| AKKGM000140 | Amuc_0113 | -1.48 | pyrimidine operon attenuation protein / uracil phosphoribosyltransferase |
| AKKGM000696 | Amuc_0594 | -1.05 | cytidylate kinase |
| AKKGM001493 | Amuc_1269 | -0.96 | thioredoxin reductase (NADPH) |
| AKKGM002379 | Amuc_2027 | -0.70 | dihydroorotase |
| AKKGM000139 | Amuc_0112 | -0.68 | aspartate carbamoyltransferase catalytic subunit |
| AKKGM000716 | Amuc_0612 | -0.54 | DNA polymerase III subunit delta' |
| pathway：Pantothenate and CoA biosynthesis | | |  |
| Metabolites：beta-Alanine、Uracil | |  |  |
| up |  |  |  |
| AKKGM001391 | Amuc_1178 | 1.27 | ketol-acid reductoisomerase |
| AKKGM000878 | Amuc_0747 | 1.17 | aspartate 1-decarboxylase |
| down |  |  |  |
| AKKGM001297 | Amuc_1097 | -1.53 | dephospho-CoA kinase |
| AKKGM002037 | Amuc_1738 | -1.02 | pantetheine-phosphate adenylyltransferase |
| AKKGM002182 | Amuc_1859 | -0.89 | 2-dehydropantoate 2-reductase |
| AKKGM000955 | Amuc_0809 | -0.66 | acetolactate synthase I/III small subunit |
| pathway：Purine metabolism | |  |  |
| Metabolites：Adenine、Hypoxanthine | |  |  |
| up |  |  |  |
| AKKGM001522 | Amuc_1298 | 2.75 | bifunctional enzyme CysN/CysC |
| AKKGM001523 | Amuc_1299 | 2.30 | sulfate adenylyltransferase subunit 2 |
| AKKGM001445 | Amuc_1228 | 1.50 | DNA polymerase III subunit gamma/tau |
| AKKGM001763 | Amuc_1505 | 0.74 | DNA-directed RNA polymerase subunit alpha |
| AKKGM001231 | Amuc_1041 | 0.69 | DNA-directed RNA polymerase subunit beta |
| AKKGM000447 | Amuc_0374 | 0.55 | DNA polymerase III subunit alpha |
| down |  |  |  |
| AKKGM001134 | Amuc_0959 | -1.46 | nucleoside-triphosphatase |
| AKKGM002295 | Amuc_1954 | -1.29 | GMP synthase (glutamine-hydrolysing) |
| AKKGM002501 | Amuc_2125 | -1.12 | adenylosuccinate synthase |
| AKKGM001105 | Amuc_0934 | -1.08 | adenylate kinase |
| AKKGM001109 | Amuc_0937 | -1.07 | exopolyphosphatase / guanosine-5'-triphosphate,3'-diphosphate pyrophosphatase |
| AKKGM002279 | Amuc_1941 | -0.95 | adenylosuccinatelyase |
| AKKGM000190 | Amuc_0155 | -0.84 | phosphomannomutase |
| AKKGM002567 | Amuc_0001 | -0.83 | ADP-ribose pyrophosphatase |
| AKKGM000716 | Amuc_0612 | -0.54 | DNA polymerase III subunit delta' |
| pathway：Arginine and proline metabolism | | |  |
| Metabolites：Spermidine、Fumarate、1-Pyrroline-2-carboxylate、Putrescine | | | |
| up |  |  |  |
| AKKGM001471 | Amuc_1252 | 2.89 | glutamine synthetase |
| AKKGM002412 | Amuc_2051 | 1.56 | glutamate dehydrogenase (NADP+) |
| down |  |  |  |
| AKKGM002342 | Amuc_1990 | -1.36 | pyrroline-5-carboxylate reductase |
| AKKGM000613 | Amuc_0528 | -0.86 | glutamate-5-semialdehyde dehydrogenase |
| AKKGM001031 | Amuc_0872 | -0.80 | acetylornithine aminotransferase |
| AKKGM000614 | Amuc_0529 | -0.80 | glutamate 5-kinase |
| AKKGM001699 | Amuc_1448 | -0.66 | glutamate N-acetyltransferase / amino-acid N-acetyltransferase |

Table S11 Primer pairs used for *qRT-PCR* in this study

| Gene number | Locus Tag | Gene/protein annotation | Primer pair |
| --- | --- | --- | --- |
| *16sDNA* | NA | Transcription | F,5'-AACGCGAAGAACCTTAC-3' |
|  |  |  | R,5'-CGGTGTGTACAAGACCC-3' |
| *Hexa-B1* | amu:Amuc_2018 | hexosaminidase | F,5'-GCCACAAGAACCGTCCAAGCC-3' |
|  |  |  | R,5'-CCACAGTGCGTTCCAGAGTATAGC-3' |
| *Hexa-B2* | amu:Amuc_1669 | hexosaminidase | F,5'-AAGTTGGAATGTAGCGGAGAAGCC-3' |
|  |  |  | R,,5'-CAGCCTCTTCACCTCCTCCTCTC-3' |
| *Hexa-B3* | amu:Amuc_2136 | hexosaminidase | F,5'-GCCGAATTACCGCCTGACCATG-3' |
|  |  |  | R,5'-CCACCTTGACCTTCTTGCCTACG-3' |
| *Hexa-B4* | amu:Amuc_2018 | hexosaminidase | F,5'-CGGCGGCATTCTGCTATCCTTC-3' |
|  |  |  | R,5'-TCCTGTCTGAGTTCCGTTCTGGAC-3' |
| *Hexa-B5* | amu:Amuc_0369 | hexosaminidase | F,5'-GCGTATAGCGAGCGTATGGATCTG-3' |
|  |  |  | R,5'-GCGTCCATGTGCCGATCCTG-3' |
| *Hexa-B6* | amu:Amuc_0868 | hexosaminidase | F,5'-CTGGCGGAAGTTGCCTGGAC-3' |
|  |  |  | R,5'-CGCGTAATGACGGCCTTCGG-3' |
| *Laz1* | amu:Amuc_0290 | beta-galactosidase | F,5'-GTTCAGCATCGCCAGCCTCATG-3' |
|  |  |  | R,5'-GTTCCGGTTGCGTACTGCCATC-3' |
| *Laz2* | bhl:Bache_0093 | beta-galactosidase | F,5'-ACAGCAATGGTTCCGTGGTGAAG-3' |
|  |  |  | R,5'-AAGCCGTGGCCGTAATAGTGTTG-3' |
| *Laz3* | amu:Amuc_1666 | beta-galactosidase | F,5'-CCGCACTTCCGTGGACGATATG-3' |
|  |  |  | R,5'-CTCCTGGCATCGTTAAGCTCCTTC-3' |
| *FucA1* | amu:Amuc_0146 | alpha-L-fucosidase | F,5'-TGGAGGAAGCAGCCGGACAG-3' |
|  |  |  | R,5'-TCAGGTCACGGTCTTCTACTGGAG-3' |
| *FucA2* | amu:Amuc_0392 | alpha-L-fucosidase | F,5'-GGAGACACCACCATCAACCATGC-3' |
|  |  |  | R,5'-CCGCCGCCACGTTCAGAATG-3' |
| *FucA3* | amu:Amuc_0846 | alpha-L-fucosidase | F,5'-GTCCGCAGTCCGCCATGTAC-3' |
|  |  |  | R,5'-AATCTCCGTCAATAGCCAGTTCCG-3' |
| *NaGlu* | tsa:AciPR4_1553 | alpha-N-acetylglucosaminidase | F,5'-TGGCGGCGGATTACAACAAGAC-3' |
|  |  |  | R,5'-AAGAGTTCAAGGCTTCCAGATGGC-3' |
| *GalA* | amu:Amuc_1187 | alpha-galactosidase | F,5'-ATATCGTGCTCAGCCTGTCCAATG-3' |
|  |  |  | R,5'-ATATGCTTCTGCCAGCGTTCCTG-3' |
| *malZ* | amu:Amuc_1870 | alpha-glucosidase | F,5'-GAGCCGAGTTATGCCGTAAGCC-3' |
|  |  |  | R,5'-TTGCTCCTCCGTGCGAATGC-3' |
